# The Association Between Emotional and Behavioral Problems in Children with Autism Spectrum Disorder and Psychological Distress in Their Parents: A Systematic Review and Meta-analysis

**DOI:** 10.1007/s10803-018-3605-y

**Published:** 2018-05-18

**Authors:** Isabel Yorke, Pippa White, Amelia Weston, Monica Rafla, Tony Charman, Emily Simonoff

**Affiliations:** 1 Institute of Psychiatry, Psychology & Neuroscience, King’s College London, 16 De Crespigny Park, London, SE5 8AF UK; 2 Department of Psychology, University of Bath, Bath, UK

**Keywords:** Autism spectrum disorder, Additional psychopathology, Emotional and behavioral problems, Mental health, Parenting stress, Psychological distress

## Abstract

This review (Prospero Registration Number: CRD42017057915) aimed to systematically identify and summarize existing research on the relationship between additional emotional and behavioral problems (EBP) in children with autism, and parenting stress (PS) and mental health problems (MHP) in their parents. Sixty-seven studies met criteria for inclusion in the review, 61 of which were included in the meta-analysis. Pooled correlation coefficients were in the low to moderate range ($\bar {r}= - .21\;{\text{to}}\;.43$). Some evidence for moderation by measurement characteristics was found. Narrative review of concurrent adjusted associations showed some evidence for shared relationships with other factors, most notably ASD severity and parent perception of own parenting. Longitudinal studies showed mixed evidence for bidirectional predictive relationships between child EBP and parent psychological distress variables.

**Electronic supplementary material:**

The online version of this article (10.1007/s10803-018-3605-y) contains supplementary material, which is available to authorized users.

## Introduction

Research in the general population has established well-characterized associations between child psychopathology and elements of parental psychological distress, including parenting stress (PS) (Crnic et al. 2005) and mental health problems (MHP) (Goodman et al. 2011). Population-based research has shown PS (Keyser et al. 2017) and MHP (Ford et al. 2004) to retain a significant association with child psychopathology after adjusting for other family, child and contextual factors. Longitudinal research has shown support for reciprocal predictive relationships between child emotional and behavioral problems (EBP) and parent psychological wellbeing, for example depression (Bagner et al. 2013) and in certain conditions, PS (Stone et al. 2016). Although it is plausible that a shared genetic component may account for the correlation between psychopathology in children and their parents, research conducted using genetically sensitive designs has evidenced an important role for environment in the intergenerational association of anxiety (Eley et al. 2015), depression (McAdams et al. 2015) and conduct problems (D’Onofrio et al. 2007).

Children with autism spectrum disorder (ASD) (American Psychiatric Association 2013) are highly likely to meet criteria for additional mental health disorders (de Bruin et al. 2007; Salazar et al. 2015; Simonoff et al. 2008). These commonly take the form of both internalizing (e.g. anxiety or depressed mood) and externalizing (e.g. conduct problems, oppositional behavior or hyperactivity) problems. They have been identified as a source of particular difficulty and unmet need for individuals and their families (Cadman et al. 2012; Kring et al. 2008). Some previous research indicates that such EBP are more closely related to family functioning (McStay et al. 2014b; Pozo et al. 2014) and parent wellbeing (Vasilopoulou and Nisbet 2016) than is core ASD symptom severity.

Increased psychological distress has also been demonstrated in parents of children with ASD. Parents of children with ASD experience high rates of PS, even in comparison to parents of children with other developmental disorders, intellectual difficulties and physical disabilities (Estes et al. 2009; Hayes and Watson 2013). Increased rates of MHP are also seen both before and after the child’s birth (Bolton et al. 1998; Jokiranta et al. 2013). Research in the general population has shown that PS and MHP are related but separable phenomena, with each tending to increase vulnerability to the other (Deater-Deckard 2004). For example, underlying susceptibility to poor mental health may compromise ability to cope with parenting demands. Equally, intense parenting challenges may initiate the development of psychopathology. In parents of children with ASD specifically, it is plausible for both these processes to be at work. Given the heightened rates of psychopathology in both children with ASD and their parents, and the potential for each to exacerbate the other, investigating their association in these families essential.

A growing body of research has investigated PS and MHP for their candidacy as contributors to, or consequences of, additional EBP in children with ASD. The purpose of this systematic review and meta-analysis is to identify and synthesize such research. Most has been cross-sectional, seeking to identify factors that account for variance in the rates and severity of parent MHP, PS or child EBP. A plethora of other child, parent and contextual factors has been investigated for their involvement in the relationships of interest, meaning that comparison across studies is not straightforward. In order to summarize such research, the current study includes a meta-analysis of unadjusted concurrent relationships and narrative review of concurrent relationships adjusted for other factors. Longitudinal research is also reviewed narratively.

This review had four main research objectives. Firstly, we aimed to establish the magnitude of the concurrent associations between child EBP and parent psychological distress variables (MHP and PS) in families of children with ASD. Based on research in the general population (Costa et al. 2006; Goodman et al. 2011; Crnic et al. 2005), meta-analysis was expected to reveal significant pooled concurrent associations of small to moderate magnitude. Although the relationships between parent psychological distress variables and child EBP appear similar in magnitude across externalizing and internalizing problems in the general population (e.g. Goodman et al. 2011), evidence nevertheless supports an etiological distinction between the two (Cosgrove et al. 2011). We therefore opted to run separate analyses for child externalizing and internalizing problems, in addition to those for total EBP.

Secondly, we aimed to explore which methodological factors explain variation across studies in the magnitude of these relationships. Goodman and colleagues (2011) found in a meta-analysis of general population studies that the relationship between maternal depression and child EBP was statistically moderated by various methodological factors including mean age of the child sample, clinical versus community recruitment of mothers and informant for child EBP. We correspondingly aimed to investigate these factors as potential moderators in our analyses. Of particular interest was the effect of informant, since the majority of the relevant literature relies on parent-report questionnaire measures for both child and parent variables. This approach may inflate associations due to shared method biases (Podsakoff et al. 2003) and the possible tendency for distressed parents to rate their children’s problems as more severe (Najman et al. 2000). We therefore planned separate analyses for effect sizes deriving from a rating of child EBP by the index parent, or those by an alternative informant.

Our third objective was to ascertain whether the associations of interest maintain once other child factors (e.g. ASD severity; IQ), parent factors (e.g. coping style; parenting behaviors; social support) and contextual factors (e.g. family income; socio-economic status) are statistically accounted for. In order to guide future mental health research in families of children with ASD, it is important to (a) establish whether other factors can fully or partially account for the relationship between parent and child psychological well-being and (b) identify other factors which may play a role in the architecture of these relationships. Although studies conducted at single time-points cannot inform us as to causality or the temporal precedence of one factor over another (Kraemer et al. 2001), they may guide us as to which variables are most likely to be involved, and therefore which should be selected for investigation in longitudinal and intervention research.

Our final objective was to examine the literature for evidence as to predictive (longitudinal) relationships between child EBP and parental MHP or PS in this population. Specifically, we wished to establish whether child EBP predicts elements of parent psychological distress, whether the opposite is true, or whether both are risk factors for each other. To meet this objective, a descriptive review was planned. Although quantitative analysis was to be performed if available data allowed it, in fact they did not. Based on research in the general population, we expected to find bidirectional predictive relationships, i.e. earlier parent MHP and PS predict later child EBP and vice versa (Bagner et al. 2013; Neece et al. 2012; Nicholson et al. 2011; Zadeh et al. 2010).

## Methods

### Search Procedures

Search terms were initially run in the Cochrane Database of Systematic Reviews to evaluate coverage of the topic of interest by existing reviews. Upon finding no equivalent reviews, the same search was then conducted in the Cochrane Library, Medline, PsycINFO, PsycArticles, Embase and Web of Science databases. Search strategies included keywords pertaining to ASD (including “autis*”, “Asperger*” and “pervasive developmental disorder”) and additional EBP (including “psychiatr*, “psychopatholog*”, “externali$ing”, “internali$ing”, “behavio$r problem*” and “emotional problem*”). These terms were combined with terms for caregiver psychological distress, produced by searching for “parent*”, “mother*”, “maternal”, “father*”, “paternal”, or “caregiver*” within two words of “stress*”, “distress*”, “psychopatholog*”, “psychiatr*”, “mental health” or “mental disorder*”. Subject headings were used according to the capabilities of each database. Complete search strategies are provided in the supplemental material. Searches were conducted on three occasions: 12th April 2016, 6th September 2016 and 9th April 2017. Email alerts were set up to capture relevant literature published after these dates. Reference lists of included studies were searched manually to identify any eligible studies missed by the search strategy. This study was added to the PROSPERO registry at the study screening stage (CRD42017057915).

### Inclusion Criteria

This review included original articles published in or after the year 2000 in the English language that met the following criteria.

#### Participant Characteristics

Study samples were required to include at least 20 individuals with a reported clinical diagnosis of ASD, and their parents or main unpaid caregivers (hereafter, parents). The minimum mean age of the children was 3 years (36 months) and the maximum mean age was 21 years. There was also a maximum upper age limit of 25 years, in order to conserve homogeneity (i.e. samples whose age range extended beyond 25 years were excluded). There were no specifications regarding the age of the parents. Studies with multiple time-points were included provided a relevant analysis was conducted for at least one time within these age boundaries. If analysis at more than one time-point met criteria, the earliest of these was included in the meta-analyses of concurrent association. In the case of multiple studies reporting on the same sample, the study that reported the most relevant information was selected for inclusion in the analysis, whilst the others were excluded.

#### Measurement Requirements

Studies were required to include at least one quantitative measurement of additional EBP in young people with diagnosed ASD. This measure was required primarily to tap common EBP. Measures containing several items pertaining to ASD-like behaviors were permitted, provided these did not form a majority. Secondly, studies were required to include at least one quantitative self-report measure of caregiver PS or common MHP, e.g. anxiety and/or depression. Quality of life measures often include a mental health component; however, this concept is not necessarily the same as MHP. Such studies have been reviewed elsewhere (Vasilopoulou and Nisbet 2016), thus they were not included here.

#### Analysis Requirements

Studies reporting results of at least one statistical analysis of association between the two required measures, within the sample of participants meeting above criteria, were eligible. To be included in the meta-analysis component, an unadjusted (zero-order) correlation coefficient was required (ideally, Pearson’s *r*). In studies that conducted a relevant analysis, but did not provide Pearson’s *r*, various procedures were used to obtain or impute an effect size. Firstly, authors were contacted and asked to supply Pearson’s *r*. Failing this, we imputed Pearson’s *r* from Spearman’s rho, phi coefficients t-tests or Chi square values using the Practical Meta-Analysis Effect Size Calculator (Wilson 2001). This was done for 5 studies (8.20%). In the case of one study (Bromley et al. 2004), effect sizes were only reported for significant associations, and those for non-significant associations were not recoverable. In such cases, a Pearson’s *r* value of .00 was imputed. Standardized regression coefficients (betas) for simple regressions (with only one predictor) were used in place of Pearson’s *r* for several studies to minimize missing data. Studies that reported only statistics for adjusted relationships (e.g. multiple regressions, partial correlations, ANCOVA) were excluded from the meta-analysis component. Such analyses were reviewed narratively to address Objective 3.

### Exclusion Criteria

Samples in which the majority of participants had non-idiopathic ASD (e.g. that attributable to a known genetic syndrome) were excluded, since such syndromes tend to have their own characteristic profiles of symptoms (Glennon et al. 2017), including EBP. In samples comprising children with various developmental disorders, those with less than 50% diagnosed with ASD were excluded. Studies in which the majority of participating children lived away from the family home were excluded.

### Study Selection and Data Extraction

Studies identified in the search were reviewed for inclusion by IY. Just over 25% of the 414 articles which progressed to screening by full text (*n* = 106) were additionally reviewed by MR. These were randomly selected but stratified by inclusion/exclusion by IY. Any disagreements were discussed and resolved by ES where necessary. IY and MR initially disagreed on 11 studies (9.43%). Of these, one was a study identified as eligible by MR, which was initially missed by IY. This was subsequently included in the descriptive synthesis of longitudinal relationships.

Data were extracted (according to the headings in Table 1) from all included studies by IY. Data were also extracted from a randomly selected 49% of studies (*n* = 30) included in the meta-analysis by PW or AW. IY and the other researchers agreed on 179 out of 208 items entered into the characteristics table (86%). Where discrepancies in data extraction were identified, these were discussed, and consensus agreed. Of the 29 discrepancies between coders, three were mistakes by the primary coder.

**Table 1:** Study characteristics

Study	Sample size; recruitment source	Child: proportion male	Child: proportion with ASD; source	Child: mean age (SD), range	Parent: proportion mothers	Parent: mean age (SD), range	Child measure (subscale)	Parent measure (subscale)	Effect size (N in analysis)	Meta-analysis number
Bader and Barry (2014)	84; community	.87	1.00; parent	11.0 (3.3), 6-16^a^	.96	43.0 (6.4), 30–56	CBCL 6–18 (ext.)	PSI-SF (PD)	–	–
Bader et al. (2015)	111; community	.86	1.00; parent	11.0 (3.5), 6–18	.97	42.0 (6.8), 25–58	CBCL 6–18 (ext.)	PSI-SF (PD)	.27 (111)	1, 2, 8
Baker et al. (2011)	149; community	.74	1.00; research	14.8 (1.9), 10–22	1.00	44.4 (5.2), NR	SIB-R GMI	CES-D	.28 (137^a^)	4
Beer et al. (2013)	28; clinic	.86	1.00; clinical	9.0 (4.3), 3–20	.86	43.2 (8.4), 32–76	NCBRF	QRS-F (PFP-5D) HADS (anx.)	.81 (28) .56 (27)	1, 8 4
Bekhet (2016)	117; community	.86	1.00; parent	10.9 (3.3), 3–17	.97	41.0 (6.0), 23–58	NCBRF	CES-D	.35^d^ (117)	4
Benson (2014)	113; community	.86	1.00; research	8.6 (1.5), NR	1.00	42.0 (5.2), NR	NCBRF	Own MHP scale	–	–
Benson and Kersh (2011)	96; community	.87	1.00; research	8.7 (1.5), NR	1.00	41.9 (5.0), NR	NCBRF	CES-D	.32 (96)	4
Brobst et al. (2009)	25; community	NR	1.00; parent	6.6 (2.7), 2–12	1.00	38.5 (6.5), 23–55	ECBI (intensity)	PSI-SF	.54 (23)	1
Bromley et al. (2004)	71; community	.80	1.00; parent	10.3^a^ (NR), ≤ 18	1.00	NR	DBC-P (disrupt.) (anx.)	GHQ-12	.30^b^ (68^c^) .00^a^ (68^c^) .00^a^ (68^c^)	4 5 6
Chu and Richdale (2009)	46; community	NR	.56; parent	7.2 (2.4), 2–12	1.00	40.2 (6.2), 28–57	SDQ	PHS (freq.) DASS-21 (anx.)	.50 (43) .27 (43)	1 4
Conner et al. (2013)	30; community	NR	1.00; research	NR (NR), 12–17	.97	NR	ABC (irrit.) CASI-20-P CASI-20-O	STAI-state	.70 (30) .38 (30) .10 (23)	4, 5 6 7
Conner and White (2014)	67; community	.82	1.00; parent	10.5^a^ (NR), 4–17	1.00	41.0 (7.2), 27–57	ABC (irrit.)	Perc. SS DASS-21	.37 (67)^e^ .42 (67)^e^	1, 2, 8 4, 5
Craig et al. (2016)	45; clinic	.66	1.00; clinical	8.3 (3.6), 3–12	1.00	41.8 (4.8), NR	CBCL 1.5-5/6–18 (ext.) (int.)	PSI-SF	.21^b^ (42) .04^b^ (42) .20^b^ (42)	1 2 3
Falk et al. (2014)	250; community	NR	1.00; parent	8.4 (3.9), 4–17	1.00	39.9 (6.3), 24–58	SDQ (cond.)	DASS-21 (stress) DASS-21 (anx.)	.31 (250) .31 (250)	1, 2, 8 4, 5
Farmer et al. (2012)	124; clinic	.85	1.00; research	7.4 (2.4), 4–14	NR	NR	ABC (irrit.)	PSI-SF PSI-SF (PD)	.39^e^ (124) .20^e^ (124)	1, 2 8
Firth and Dryer (2013)	109; community	.80	1.00; parent	7.9 (2.4), 4–12	NR	NR	NCBRF	PSS DASS-21 (anx.)	.30 (109) .44 (109)	1, 8 4
Fitzgerald et al. (2002)	100; population	.76	1.00; parent	13.6 (NR), 2–25	1.00	45.0 (NR), 27–67	VABS-MBD (Part 1)	GHQ-30	.29 (100)	4
Foody et al. (2014)	74; NR	.77	1.00; clinical	8.9 (3.6), 2–17	1.00	41.2 (6.0), 26–65	CPRS (oppos.)	PSI-SF HADS PSI-SF (PD)	.60 (51)^e^ .20 (50)^e^ .36 (51)^e^	1, 2 4, 5 8
Fung et al. (2015)	91; community	.82	1.00; parent	13.4 (5.1), 7–25	.90	44.3 (7.3), 31–62	GDS-CS	K6	.43 (91)	4
Gallagher et al. (2008)	32; community	NR	.66; parent	11.5 (3.4), 3–19	.75	42.8 (5.8), NR	SDQ	HADS (anx.)	.17^d,e^ (32)	4
Giovagnoli et al. (2015)	130; clinic	.85	1.00; research	3.5 (.8), NR	NR	37.7 (2.2), NR	CBCL 1.5-5 (ext.) (int.)	PSI-SF	.28 (130)^e^ .30 (130)^e^ .38 (130)^e^	1 2 3
Hall and Graff (2012)	70; community	.83	1.00; parent	8.7 (4.2), 3–21	.69	40.0 (8.5), NR	MBI (ext.) (int.)	PSI-SF	.46 (70) .21 (70) .55 (70)	1 2 3
Hastings and Brown (2002)	26; community	.65	1.00; clinical	12.2 (2.5), NR	1.00	41.0 (5.0), NR	DBC-T	HADS (anx.)	.54^d^ (26)	7
Hastings et al. (2005)	48; clinic	.85	1.00; research	3.1 (.37), 2.3–3.8	1.00	34.5 (4.1), NR	DBC-P (mother) DBC-P (father)	QRS-F (PFP-5D) HADS (anx.)	.59 (48) .32 (48) .02 (41)	1 4 7
Hastings et al. (2014)	60; community	.80	1.00; parent	9.8 (2.3), 4–15	1.00	42.1 (4.8), 28–52	SDQ	HADS (dep.)	.24 (60)^e^	4
Herring et al. (2006)	79; clinic	.89	1.00; research	3.1 (.6), 1–4	1.00	NR	DBC-P	GHQ-28	.37 (78)^e^	4
Huang et al. (2014)	52; clinic	.94	1.00; clinical	6.3 (2.3), 3–12	.87	39.2 (5.9), NR	Chinese SDQ (Cond.) (Emot.)	Chinese PSI-SF PSI-SF (PD)	.43 (52)^e^ .49 (52)^e^ .25 (52)^e^ .23 (52)^e^	1 2 3 8
Jellett et al. (2015)	97; community	.85	1.00; parent	4.3 (1.1), 1–5	.91	36.1 (5.5), NR	DBC-P24	DASS-21 (stress) (dep.)	.39 (97) .31 (97)	1, 8 4
Jones et al. (2014)	71; community	.83	1.00; parent	13.0 (2.3), 7–16	1.00	45.0 (4.6), NR	SDQ	HADS (anx.)	.29^d^ (70)	4
Kerns et al. (2015)	59; community	.78	1.00; research	10.6 (2.8), 7–17	NR	NR	BASC-2 (dep.)	PSI/SIPA (child age dependent)	.66 (48)	1, 3
Kim et al. (2016)	234; community	.82	1.00; parent	7.1 (3.4), 2–19	1.00	37.5 (7.1), NR	Own EBP scale	CES-D BSF	.35 (234)	4
Krakovich et al. (2016)	79; community	NR	1.00; research	5.9 (1.6), 3–9	.91	NR	BASC-2 BSI	PSI (Parent Domain)	.42 (75)	1, 8
Lancaster et al. (2014)	27; community	.82	.55; parent	7.0 (2.1), 4–9	1.00	35.0 (5.7), 25–44	BPI-01 (frequency)	HADS (dep.)	.55^e^ (27)	4
Lecavalier et al. (2006)	293; community	.83	1.00; parent	9.0 (3.4), 3–18	.86	39.9 (7.1), NR	NCBRF (cond.) (insec/anx.)	PSI-SF	.40^b^ (253) .17^b^ (253)	1, 2 3
Lee and Chiang (2017)	138; community	.75	1.00; clinical	15.8 (1.9), 10–19	1.00	45.1 (3.9), NR	Korean VABS-2 MBI (dichotomized)	Korean SIPA	.46^h^ (138)	1
Lovell and Wetherell (2016)	118; community	NR	1.00; parent	9.8 (4.4), 3–19	.94	41.3 (7.6), 23–63	SDQ	Perc. SS	.28 (118)	1
Machado et al. (2016)	102; community	.84	1.00; clinical	10.3 (5.3), 3–21	.82	40.6^a^ (6.6^a^), 26–62	ABC	HADS	.26^g^ (102)	4
Manning et al. (2011)	195; community	.83	1.00; parent	8.8 (2.1), 6–12	.96	40.9 (6.1), 24–54	CBCL	PSI-SF (PD)	.39 (195)	1, 8
McStay et al. (2014a)	150; community	.87	1.00; clinical	13.2 (3.0), 6.−18	.72	NR	DBD (cond.)	Dutch PSI-SF (PD)	.19 (150)	1, 2, 8
McStay et al. (2014b)	98; community	.86	1.00; parent	8.9 (3.8), 3–16	1.00	41.8 (6.0), NR	BASC-2 (ext.) (int.)	PSI-SF (PD)	.36 (98) .11 (98)	1, 2, 8 3
Osborne and Reed (2009)(1)	65; community	.91	1.00; clinical	3.3 (.8), 2–4	NR	NR	CPRS	QRS-F	–	–
Osborne and Reed (2009)(2)	72; community	.97	1.00; clinical	8.7 (3.5), 5–16	NR	NR	SDQ	PSI	–	–
Pakenham et al. (2005)	47; community	.85	1.00; parent	10.8 (NR), 10–12	1.00	NR	ECBI (intensity)	DASS-21 (anx.)	.31 (47)	4
Park et al. (2013)	56; clinic	.86	1.00; clinical	9.4 (2.0), 6–13	1.00	NR	Korean CBCL (ext.) (int) STAIC-S (child self-report)	STAI-S	.47 (56) .48 (56) .42 (56) .26 (56)	4 5 6 7
Paynter et al. (2013)	43; community	.84	1.00; parent	4.1 (.8), 2–6	.58	37.4 (7.9), NR	SDQ	PSI-SF DASS-21 (anx.) DASS-21 (stress)	.65 (41) .16 (41) .30 (41)	1 4 8
Peters-Scheffer et al. (2012)	104; community	.75	1.00; research	5.5^a^ (NR), 2–9	1.00	NR	CBCL 1.5-5 (ext.) (int.)	Dutch PSI-SF	.55 (102) .51 (102) .49 (102)	1 2 3
Reaven et al. (2015)	31; clinical	.74	1.00; research	13.8 (3.0), 7–18	.84	44.4 (7.4), 30–75	SCARED-P	STAI-state	.21 (31)	4, 6
	32; community	NR	1.00; clinical	3.5 (.6), > 5	1.00	NR	DBC-P	QRS-F	–	–
Reed et al. (2016)	93; community	.83	1.00; parent	10.5 (4.1), 3–18	1.00	43.3 (6.8), 25–58	SDQ	QRS-F	.28 (93)	1
Rezendes and Scarpa (2011)	134; community	.80	1.00; parent	9.2 (NR), 3–16	1.00	39.0 (8.0), NR	SDQ	QRS-F DASS (DA) QRS-F (PFP)	.27 (133) .06 (133) .06 (133)	1 4 8
Robinson and Neece (2015)	44; community	.71	.89; parent	3.4 (1.0), 2–5	.77	35.2 (8.5), NR	CBCL 1.5-5 (ext.) (int.)	PSI-SF (PD)	.51 (44) .48 (44) .41 (44)	1, 8 2 3
Salazar et al. (2015)	101; population	.82	1.00; research	6.7 (1.2), 4–9	.94	NR	DBC-P (disrupt.) (anx.) DBC-T	K10	.25 (86)^e^ .19 (86)^e^ .03 (86)^e^ .36 (78)^e^	4 5 6 7
Sawyer et al. (2010)	216; community	.88	1.00; clinical	11.0 (2.9), 6–17	1.00	NR	SDQ	GHQ-30	.23 (216)	4
Shawler and Sullivan (2017)	130; community	.89	1.00; clinical	8.6 (2.4), 3–11	.92	39.8 (6.6), 24–58	ECBI (intensity)	PSI-SF	.72 (128) .44 (128)	1 8
Simonoff et al. (2013)	158; population	.90	1.00; research	11.7 (.9), 10–13	NR	NR	SDQ-P (cond.) (emot.)	PSI-SF	.33 (127) .30 (127) .25 (127)	1 2 3
								GHQ-12	.14 (125) .06 (125) .30 (125)	4 5 6
							SDQ-T		.01 (108)	7
							SDQ-P	PSI-SF (PD)	.19 (127)	8
Skokauskas and Gallagher (2012)	67; community	.88	1.00; research	12.7 (2.9), NR	NR	48.9 (6.2), NR	CBCL 6–18 (ext.) (int.)	BSI-GSI	.13 (67) .06 (67)	4, 5 6
Stoppelbein et al. (2016)	45; clinic	.80	1.00; research	8.7 (2.2), 6–12	.84	NR	CBCL 6–18 (ext.) (int.)	HSCL	.37 (45) .50 (45)	4, 5 6
Suzuki et al. (2015)	405; clinic	NR	.69; clinical	10.2 (3.5), 3–18	1.00	41.6 (5.4), 28–54	Japanese SDQ	Japanese GHQ-12	.22 (313)	4
Taylor and Warren (2012)	75; clinic	.88	1.00; research	5.1^a^ (NR), NR	1.00	NR	CBCL (ext.) (int.)	CES-D	.45 (75) .34 (75) .39 (75)	4 5 6
Totsika et al. (2013)	132; population	.82	1.00; parent	3.5^a^ (NR), 3–3	1.00	31.5^a^ (NR), NR	SDQ	K6	.14 (132)	4
Valicenti-McDermott et al. (2015)	50; clinical	.94	1.00; clinical	8.8 (3.0), 2–18	1.00	38.0 (7.0), NR	ABC	PSI-SF	.32 (50)^i^	1
Walsh et al. (2013)	132; community	.77	1.00; parent	9.3 (4.9), 2–18	1.00	NR	ABC	PDH (intensity)	.62 (132)	1
Warfield et al. (2014)	74; clinic	.82	1.00; clinical	6.8 (1.2), 4–9	NR	NR	Observational assessment (ext.)	PSI-SF (PD)	–	–
Weiss et al. (2012)	228; community	.82	1.00; parent	11.8 (3.6), 6–21	.93	NR	NCBRF	K6	.39 (228)	4
Weiss et al. (2015)	101; community	.75	1.00; parent	14.5 (2.1), 12–21	1.00	NR	NCBRF (insec/anx.)	PSS	.41 (101)	1, 3, 8
Weitlauf et al. (2014)	70; clinic	.88	1.00; clinical	5.0 (NR), NR	1.00	NR	CBCL (ext.) (int.)	PSI-SF	.54 (70) .50 (70) .40 (70)	1 2 3
Zaidman-Zait et al. (2014)	184; clinic	.84	1.00; research	3.0 (.6), 2–3	1.00	NR	CBCL 1.5-5	PSI-SF (PD)	–	–
Zaidman-Zait et al. (2017)	283; clinic	.84	1.00; research	3.2 (.7), 2–4	1.00	35.4 (5.4), 20–48	CBCL 1.5-5 (ext.) (int.)	PSI-SF (PD & GD composite)	.36 (283) .33 (283)	1, 2, 8 3

The meta-analysis numbers correspond to the analyses (presented in Table 2) in which each effect size was included

*NR* not reported, “–” no effect size eligible for meta-analysis (study included in narrative syntheses only). Measures are full-scale composites unless a subscale is specified

Child measures: *ABC* Aberrant Behavior Checklist, *BASC2* Behavior Assessment System for Children (Second Edition), *BPI-01* Behavior Problems Inventory *CASI-20-P[O]* 20-item Childhood Anxiety Sensitivity Index-Parent[Observer] version, *CBCL 1.5-5[6–18]* Child Behavior Checklist (for 1.5-5[6–18] year olds), *CPRS* Conners’ Parent Rating Scale, *DBC-P[T]* Developmental Behavior Checklist-Parent[Teacher] version, *DBC-P24* 24-item DBC-P, *DBD* Disruptive Behavior Disorders rating scale, *ECBI* Eyberg Child Behavior Inventory, *GDS-CS* Glasgow Depression Scale-Carer Supplement, *MBI* Maladaptive Behavior Index, *NCBRF* Nisonger Child Behavior Rating Form, *SDQ* Strengths and Difficulties Questionnaire, *SIB-R GMI* Scales of Independent Behavior-Revised General Maladaptive Index, *VABS-MBD* Vineland Adaptive Behavior Scales-Maladaptive Behavior Domain

Child subscales: *cond* conduct problems subscale, *disrupt* disruptive behavior subscale, *emot* emotional symptoms subscale *ext* externalizing subscale, *insec*/*anx* insecure/anxious subscale, *int* internalizing subscale, *irrit* irritability subscale, *oppos* oppositional Behavior subscale

Parenting stress measures: *PHS* Parenting Hassles Scale, *Perc SS* Perceived Stress Scale, *PSI-SF* Parenting Stress Index-Short Form, *PSS* Parenting Stress Scale, *QRS-F* Questionnaire on Resources and Stress-Friedrich Shortform

Parenting stress subscales: *PD* Parental Distress, *PFP* Parent and Family Problems, *PFP-5D* Parent and Family Problems-minus five depression items

Subscales: *anx* anxiety, *dep* depression

Parent MHP measures: *CES-D* Center for Epidemiologic Studies Depression Scale, *CES-D BSF* CES-D Boston Short Form, *DASS-21* 21-item Depression, Anxiety and Stress Scale, *GHQ-12[28]* 12[28]-item General Health Questionnaire,; *HADS* Hospital Anxiety and Depression Scale, *STAI* State-Trait Anxiety Inventory

^a^Estimate based on information given in the paper

^b^Converted from Spearman’s rho value

^c^Assumed that parents contributed one data-point each

^d^Unadjusted standardized regression coefficient (beta)

^e^Data sent by author

^f^Studies reporting on the same sample

^g^Converted from phi coefficient

^h^Converted from *t*-test

^i^Converted from Chi square

**Table 2:** Pooled effect sizes

	Parenting stress	Parent mental health problems	Parenting stress—refined measure criteria
Total difficulties			
Meta-analysis number	1	4	8
Pooled *r* (95% CI)	.43*** (.38–.48)	.30*** (.26–.34)	.34*** (.28–.39)
*k*	35	35	22
*N*	3625	3458	2430
*Q*	106.81***	51.38*	48.7***
*I*^*2*^	69.77%	30.64%	54.81%
Egger’s regression test (*z*)		1.72^†^	2.81**
Trim and fill *n* missing studies		3	0
Pooled *r* after trim and fill		.30*** (.25–.33)	N/A
Fail-safe *N*			2206
Externalizing			
Meta-analysis number	2	5	
Pooled *r* (95% CI)	.36*** (.31–.40)	.29*** (.17–.40)	
*k*	17	11	
*N*	2024	919	
*Q*	27.14*	28.77**	
*I*^*2*^	33.37%	68.49%	
Internalizing			
Meta-analysis number	3	6	
Pooled *r* (95% CI)	.36*** (.27–.44)	.25*** (.12–.37)	
*k*	13	9	
*N*	1420	583	
*Q*	34.53***	19.14*	
*I*^*2*^	67.14%	59.22%	
Total difficulties—alternative rater			
Meta-analysis number	N/A^a^	7	
Pooled *r* (95% CI)		.21* (.04–.37)	
*k*		6	
*Q*		11.72*	
*N*		332	
*I*^*2*^		55.92%	

*k* number of effect sizes included; *N* total number of individuals

^†^*p* < .10; *p < .05; **p < .01; ***p <. 001

^a^Analysis not performed due to low number of eligible effect sizes (*k* = 3)

### Statistical Analyses

Whether authors have designated a child or parent factor as the outcome variable, cross-sectional studies cannot provide evidence for relationships over time. Therefore, single time-point analyses are undifferentiated according to whether the child or parent variable was the nominated outcome.

Two sets of separate meta-analyses were planned. One set included studies reporting effect sizes pertaining to PS (analyses 1–3) and the other to parental MHP (analyses 4–7). Within each set, one analysis was planned for each of: total child EBP (analyses 1 and 4); externalizing problems (analyses 2 and 5); internalizing problems (analyses 3 and 6); total EBP reported by an alternative informant (analysis 7). The alternative informant had to be someone other than the parent who completed the self-report PS or MHP measure, for example, the child’s other parent, a teacher or the child themselves. The alternative informant analysis was not run for its association with PS, since only 3 studies provided eligible effect sizes. A final (post-hoc) analysis (analysis 8) was performed for the association between total child EBP and PS measures that did not include a subscale primarily rating aspects of child behavior.

### Choice of Effect Size

In order to ensure independence of data points, only one effect size per study was selected for each meta-analysis. A set of selection rules were established a priori, for cases in which studies provided multiple eligible effect sizes. Firstly, where these corresponded to multiple parental respondents, the effect size for mothers was selected because they represented the majority of respondents in most studies.

In cases where multiple effect sizes arose from use of more than one measure of a concept of interest, more widely-used and better-validated instruments were chosen. Frequency scores were favored over intensity or severity ratings. For entry into the child total problems analyses, a total EBP score was preferred. When this was unavailable, an externalizing (rather than internalizing) subscale score was entered. These were selected because in included studies in which effect sizes for externalizing, internalizing and total score were available, effect sizes based on externalizing scores were better statistical predictors of those for total score (*β* = .62 for externalizing and *β* = .46 for internalizing).

For parent measures, a total score on any given PS or MHP instrument was preferred, since these were the most widely reported and tend to be based on more items. In the absence of a total PS score, subscales pertaining to parental perception of own stress levels were preferred over those pertaining to child behavior or other potential *sources* of stress. For parental MHP, in the absence of a total MHP score, where either an anxiety or a depression scale was available, an anxiety scale was selected. From existing research, it was unclear whether anxiety or depression ratings were more representative of total MHP. However, in our analysis, meta-regression was used to test whether the type of scale used (anxiety, depression or total MHP) statistically moderated effect size.

### Calculation of Pooled Effect Sizes

Statistical analysis was performed with R statistical software version 3.2.4. (R Core Team 2013) using the *Metafor* package (Viechtbauer 2010). Raw effect sizes were transformed to Fisher’s *z* before synthesis in order to stabilize variance (Borenstein et al. 2009). Along with corresponding sample sizes, these were entered into random effects models in order to estimate pooled effect sizes. These were converted back to Pearson’s *r* scale for interpretation. Random effects models were used, due to the known differences between studies in participant characteristics and measures. This meant that sample statistics from different studies were likely to be estimates of varying population parameters. Heterogeneity across studies was examining using the *Q* statistic and *I*^2^. *I*^2^ values of ~25, ~50, and ~75% were interpreted as low, moderate, and high, respectively (Higgins et al. 2003). The analyses were then rerun with mean child age, recruitment source and measurement characteristics entered as potential moderators in order to assess their ability to explain between-study variance. Egger et al. (1997) regression test was used to statistically test for funnel plot asymmetry (association between study size and effect size), which may be indicative of publication bias. In the presence of significant funnel plot asymmetry, we planned to apply the trim and fill procedure (Duval and Tweedie 2000) to correct for upward bias of pooled effect size.

### Analysis of Adjusted Concurrent Relationships

Studies reporting analyses of relationships between the child and parent variables of interest whilst adjusting for other variables were examined in pursuit of Objective 3. Such analyses included partial and semi-partial correlations between child EBP and either parent MHP or PS, controlling for other variables. Multiple regressions and ANCOVAs with child EBP, parent MHP or PS as the dependent variable (DV) were also relevant. Analyses in which child EBP was the DV were required to include parent MHP or PS as an independent variable (IV) alongside one or more other variables. Analyses in which parent MHP or PS was the DV were required to include child EBP as an independent variable alongside one or more other factors. If child EBP and parent psychological distress share a unique relationship, significant concurrent relationships should be seen despite adjustment for other variables. Other variables appearing in two or more analyses were also assessed for their contribution to the DV, and whether this appeared to be independent of the relationship of interest.

### Analysis of Longitudinal Relationships

Studies that analyzed the relationship between child EBP and parent MHP or PS across time were reviewed descriptively in order to fulfil Objective 4. Eligible analyses included cross time correlations, regressions and Structural Equation Modelling (SEM) techniques. If child additional EBP and parent psychological distress influence each other over time, significant predictive relationships should be seen such that earlier child EBP predicts later (and/or change over time in) parent psychological distress and vice versa.

## Results

### Systematic Search

**Fig. 1:**
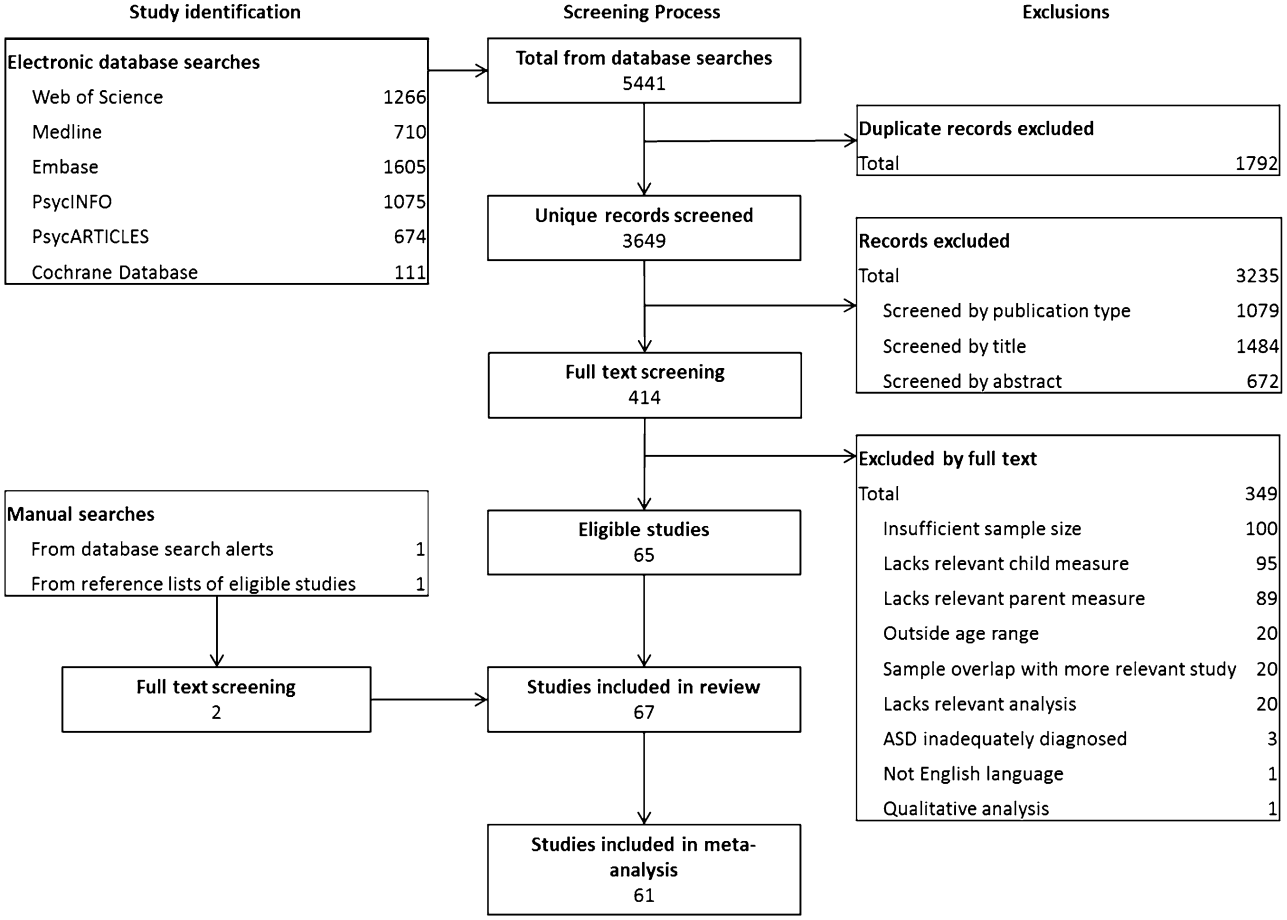
Search results and screening process

Figure 1 shows the search and screening process. Electronic database searches identified 5441 papers, of which 3649 remained after removal of duplicate records. After screening by publication type, title and abstract, 414 publications remained. For these studies, full text was retrieved and assessed for eligibility. Sixty-five publications met criteria for review. The most common reasons for exclusion at the full text stage were insufficient sample size and lack of an adequate parent or child measure. An additional two articles were identified and checked for eligibility through manual searching of email alerts and reference lists of eligible studies. Sixty-one included studies were eligible for entry into the meta-analysis after any necessary additional data had been provided by study authors. Study characteristics are reported in Table 1.

### Meta-Analyses of Single-Time Point Studies (Objectives 1 and 2)

Table 2 provides the total number of effect sizes (*k*), number of independent participants (*N*) and pooled correlation coefficients for all analyses. For analyses 1–6 (for which the same parent reported on both their own and their child’s psychological well-being), these show significant average associations of low to moderate strength (.25 ≤ $\bar {r}$ ≤ .43, CIs [.12–.49]). Within each parent factor, estimates were very similar across analyses for externalizing and internalizing difficulties, and confidence intervals overlapped (PS-externalizing $\bar {r}$ = .36, CI [.31–.40]; PS-internalizing $\bar {r}$ = .36, CI [.27–.44]; MHP-externalizing $\bar {r}$ = .29, CI [.17–.40]; MHP-internalizing $\bar {r}$ = .25, CI [.12–.37]). Looking across parent factors, PS showed a significantly stronger pooled association with total child EBP than did parent MHP, as shown by non-overlapping confidence intervals (PS-total EBP $\bar {r}$ = .43, CI [.38–.49]; MHP-total EBP $\bar {r}$ = .30, CI [.26–.34]).

*Q* and *I*^*2*^ statistics showed most analyses to have significant between-study heterogeneity, in the moderate range. Study heterogeneity was investigated more closely for Analyses 1 and 4, since these provided most power to detect factors which may account for variation between studies. Heterogeneity in the PS-total EBP analysis was particularly high (*Q* = 106.32, *p* < .001; *I*^*2*^ = 70.73%), whereas the studies forming the MHP-total EBP analysis showed lower but still significant heterogeneity (*Q* = 51.38, *p* = .03; *I*^*2*^ = 30.64%).

In the PS-total EBP analysis, mean child age was found not to account for variance effect size for either PS or MHP (*b* = − .06, *p* = .30). Recruitment source was also found not to significantly explain variance in effect size (*b* = .02; *p* = .72). It was noted, however, that several influential studies (of large effect and sample size) had used a Parenting Stress Index (PSI) total score as the measure of PS (Kerns et al. 2015; Paynter et al. 2013; Shawler and Sullivan 2017). This includes the “Difficult Child” subscale, which predominantly comprises ratings of children’s behavior problems. The possibility that overlap in content between child EBP measures and PS measures including ratings of child behavior had inflated associations between the two, was therefore investigated.

A binary variable was created denoting whether the measure used included a subscale or full scale in which parents primarily rated child characteristics (1) as opposed to rating their own psychological responses to parenting (0). When entered into a meta-regression, the effect of this factor accounted for 13.64% of heterogeneity between studies, but was not significant (*b* = .11; *p* < .09). Subset analysis showed a lower average correlation for studies coded as 0 ($\bar {r}$= .38, CI [.31–.42], *k* = 15) than for those coded as 1 ($\bar {r}$ = .47, CI [.40–.54], *k* = 20), though confidence intervals overlapped. This suggested that the original pooled estimate for the magnitude of the association between PS and child EBP may have been inflated by the conceptual overlap in some studies. Many studies reporting an effect size for a PS scale including child characteristics, also reported one for a subscale not including them. A further meta-analysis was run in which these were replaced (analysis 8). This analysis showed a somewhat reduced, but still significant, pooled effect size ($\bar {r}$= .34, CI [.28–.39]).

Heterogeneity in the MHP-total EBP analysis was not explained by child age (*b* = .01, *p* = .90). We also tested whether the scale or subscale of the parent MHP measure used could explain heterogeneity. Whether studies used a total score, an anxiety subscale or a depression subscale did not appear to be related to effect size (*b* = .01, *p* = .84). Recruitment source (clinic, community or population) was not a significant explainer of heterogeneity (*b* = .07; *p* = .08). Subset analyses revealed that pooled *r* for population-derived samples was .20 (CIs [.11–.29]; *k* = 4). For community samples $\bar {r}$ = .31 (CIs [.27–.36]; *k* = 25) and for clinical samples $\bar {r}$ = .36 (CIs [.23–.47]; *k* = 6).

Among studies measuring parent MHP, six provided an effect size of association with child EBP rated by someone other than the parent reporting own MHP. These were entered into a separate meta-analysis (analysis 7), which showed a lower, but still significant pooled magnitude of association ($\bar {r}$= .21, CI [.04–.37]). Heterogeneity for this analysis was moderate (*I*^*2*^ = 55.92%), which may reflect the range of alternative raters providing data (e.g. another parent, teacher, child self-rating). However, this could not be tested given the small number of studies. For PS, only three studies provided alternative rater EBP scores, which was deemed insufficient to conduct a meaningful meta-analysis.

Potential publication bias was assessed in the PS-total EBP and MHP-total EBP analyses. For the MHP-total EBP analysis, Egger’s regression test showed no significant evidence for funnel plot asymmetry (*z* = 1.72, *p* = .08). The trim and fill procedure imputed 3 “missing” studies; however, the pooled estimate remained largely unchanged, (see Table 2). Assessing publication bias in the total EBP-PS analysis was problematic, since conventional tests are unsuitable in the presence of substantial heterogeneity (Peters et al. 2010). For this reason, publication bias was assessed in analysis 8, in which heterogeneity was somewhat reduced. Here, there was significant evidence for funnel plot asymmetry (*z* = 2.81, *p* < .001). However, performing the trim and fill procedure did not impute any “missing” studies, possibly due to the still-considerable heterogeneity, even among large studies. Instead, we assessed tolerance for unpublished null results using Rosenthal’s (1979) fail-safe *N* procedure. This showed that an additional 2206 non-significant effect sizes would be required to reduce the pooled association such that the *p*-value reaches the .05 threshold.

### Adjusted Single Time-Point Associations (Objective 3)

Thirty-one studies provided data relevant to Objective 3. Across studies, numerous additional parent, child and contextual variables were included in multivariate analyses alongside the IV of interest, potentially sharing its relationship with the DV. In order to synthesize findings, analyses were split into those relevant to the PS-EBP relationship and those relevant to the MHP-EBP relationship. The impact of additional variables that were included in two or more analyses in either set was examined. It was noted firstly, whether the relationship of interest remained significant whilst adjusting for other variables. Secondly, it was noted which variables made a significant independent contribution to the DV. This information is presented for the PS-EBP relationship in Table 3 and for the MHP-EBP relationship in Table 4. Common additional variables included child ASD severity, family financial situation (income or SES), social support, family functioning and parents’ perception of their own parenting (e.g. parenting self-efficacy or endorsement of positive/negative parenting practices). In the majority of analyses, the relationships of interest remained significant, despite accounting for additional child, parent and contextual variables. However, this was not universally true. Important additional factors are now discussed with reference to their ability to statistically account for the relationships of interest and their independent contributions to variance in the DV.

**Table 3:** Single time-point analyses of PS-EBP relationship, adjusted for other factors

Study	N	Analysis	PS	EBP	Child					Parent					Family/context		
					Age	Sex	ASD severity	IQ	Adaptive behavior	Age	Sleep quality	Coping mechanisms	Mindfulness	Perception of own parenting	Family functioning	Social/family support	Income/SES
Zaidman-Zait et al. (2017)	283	HMR	DV	**	–	–	n.s.	–	–	–	–	**	–	–	***	***	n.s.
Lecavalier et al. (2006)	253	HMR	DV	*	–	–	C	–	–	–	–	–	–	–	–	–	–
Falk et al. (2014)	250	SMR	DV	n.s.	–	–	*	–	–	**	–	–	–	**	–	**	–
Manning et al. (2011)	195	HMR	DV	**	–	–	n.s.	–	–	*	–	***	–	–	–	n.s.	n.s.
McStay et al. (2014a)	150	HMR	DV	*	n.s.	–	n.s.	n.s.	–	–	–	–	–	–	–	–	–
Rezendes and Scarpa (2011)	140	MR	DV	**	–	–	C	–	–	C	–	–	–	–	–	–	–
Lee and Chiang (2017)	138	SMR	DV	***	*	*	–	–	–	–	–	–	–	–	–	–	–
Giovagnoli et al. (2015)	130	SMR	DV	*	–	–	n.s.	n.s.	n.s.	–	–	–	–	–	–	–	–
Bader et al. (2015)	111	HMR	n.s.	DV	n.s.	–	***	–	–	n.s.	–	–	–	**	–	–	n.s.
Firth and Dryer (2013)	109	HMR	DV	n.s.	n.s.	n.s.	*	–	–	–	–	–	–	–	–	–	–
Weiss et al. (2015)	101	HMR	**	DV	n.s.	n.s.	n.s.	–	–	–	–	–	–	–	–	–	n.s.
McStay et al. (2014b)	98	HMR	DV	*	n.s.	–	–	–	–	–	–	n.s.	–	–	n.s.	*	–
Osborne and Reed (2009) (2)	83	SPC	DV	***	–	–	C	C	C	–	–	–	–	–	–	–	–
Warfield et al. (2014)	74	HMR	DV	n.s.	–	–	–	–	–	–	–	–	–	–	–	**	–
Hall and Graff (2012)	70	MR	DV	***	–	–	–	–	–	–	–	–	–	–	–	n.s.	–
Osborne and Reed (2009) (1)	65	SPC	DV	n.s.	–	–	C	C	C	–	–	–	–	–	–	–	–
Jones et al. (2014)	61	HMR	DV	***	–	–	–	–	–	–	–	–	n.s.	–	–	–	*
Huang et al. (2014)	52	MR	DV	n.s.	–	–	n.s.	–	–	–	–	–	–	–	–	–	–
Foody et al. (2014)	51	HMR	DV	*	–	–	*	–	n.s.	–	n.s.	–	–	–	–	–	–
Kerns et al. (2015)	48	ANC	**	DV	–	–	C	–	–	–	–	–	–	–	–	–	–
Chu and Richdale (2009)	46	SMR	DV	***	–	–	–	–	–	–	***	–	–	–	–	–	–
Hastings et al. (2005)	41	HMR	DV	***	–	–	n.s.	–	n.s.	–	–	–	–	–	–	–	–
Beer et al. (2013)	28	PC	DV	***	–	–	–	–	–	–	–	–	C	–	–	–	–

Studies are presented in order of size (descending). For each study, the dependent variable (DV) is denoted and other variable are treated as independent variables (IV). Their contribution to final model is denoted by its significance (n.s. non-significant; ^†^*p* < .1; *p < .05; **p < .01; ***p < .001; ****p < .0001). Variables of interest not included as IV in individual studies are denoted with –. Types of analysis are coded as: *MR* multiple regression (variables entered simultaneously or type not specified); *SMR* stepwise multiple regression; *HMR* hierarchical multiple regression; *PC* partial correlation; *SPC* semi-partial correlation; *LMR* logistic multiple regression; *C* controlled variable

**Table 4:** Single time-point analyses of MHP-EBP relationship, adjusted for other factors

Study	N	Analysis	MHP	EBP	Child				Parent						Family/context		
					Age	Sex	ASD severity	IQ	Age	sleep	Mindfulness/acceptance	PS/burden	Perception of own parenting	Stressful life events	Marital Relationship	Social/Family Support	Income /SES
Suzuki et al. (2015)	313	MR	DV	*	–	–	–	–	–	–	–	–	*	–	–	*	–
Falk et al. (2014)	250	SMR	DV	n.s.	–	–	***	–	***	–	–	–	***	–	–	–	–
Weiss et al. (2012)	228	HMR	DV	**	n.s.	n.s.	n.s.	–	–	–	***	–	–	n.s.	–	–	n.s.
Sawyer et al. (2010)	215	HMR	DV	*	n.s.	–	–	–	–	–	–	–	–	–	–	**	–
Firth and Dryer (2013)	109	HMR	DV	**	n.s.	n.s.	n.s.	–	–	–	–	–	–	–	–	–	–
Benson and Kersh (2011)	96	HMR	DV	*	–	–	–	–	–	–	–	–	–	*	*	*	n.s.
Fung et al. (2015)	82	HMR	***	DV	*	–	–	†	–	–	–	–	–	n.s.	–	–	*
Taylor and Warren (2012)	75	MR	DV	n.s.	–	–	–	–	–	–	–	–	–	–	–	n.s.	*
Machado et al. (2016)	102	LMR	DV	**	–	*	–	–	–	–	–	–	–	–	–	–	–
Weitlauf et al. (2012)	70	HMR	DV	n.s.	–	–	n.s.	–	–	–	–	***	–	–	*	–	n.s.
Bromley et al. (2004)	68	LMR	DV	*	–	–	–	–	–	–	–	–	–	–	–	*	–
Jones et al. (2014)	65	HMR	DV	n.s.	–	–	–	–	–	–	***	–	–	–	–	–	–
Park et al. (2013)	56	HMR	DV	***	*	–	–	*	–	–	–	–	–	–	–	–	–
Pakenham et al. (2005)	47	HMR	DV	n.s.	–	n.s.	–	–	n.s.	–	–	n.s.	–	**	–	†	n.s.
Chu and Richdale (2009)	46	SMR	DV	n.s.	–	–	–	–	–	***	–	–	–	–	–	–	–
Stoppelbein et al. (2016)	45	HMR	n.s.	DV	n.s.	–	–	–	–	–	–	–	–	–	–	–	–
Gallagher et al. (2008)	32	SMR	DV	n.s.	–	–	–	–	–	–	–	*	–	–	–	**	–
Beer et al. (2013)	28	PC	DV	***	–	–	–	–	–	–	C	–	–	–	–	–	–
Hastings and Brown (2002)	26	HMR	DV	n.s.	–	–	–	–	–	–	–	–	**	–	–	–	–

Studies are presented in order of size (descending). For each study, the dependent variable (DV) is denoted and other variable are treated as independent variables (IV). Their contribution to final model is denoted by its significance (n.s. non-significant; ^†^*p* < .1; *p < .05; **p < .01; ***p < .001; ****p < .0001). Variables of interest not included as IV in individual studies are denoted with –. Types of analysis are coded as: *MR* multiple regression (variables entered simultaneously or type not specified); *SMR* stepwise multiple regression; *HMR* hierarchical multiple regression; *PC* partial correlation; *SPC* semi-partial correlation; *LMR* logistic multiple regression; *C* controlled variable

#### Child Factors

Child age, sex, IQ and adaptive behavior very rarely showed association with the DV. Where they did, the relationship of interest (EBP-PS or EBP-MHP) was also independently significant. Therefore, these child factors did not appear to account for the associations of interest to any important extent. Several studies report the PS-EBP relationship to remain significant despite accounting for ASD severity, which itself was not related to the dependent variable (Giovagnoli et al. 2015; Hastings et al. 2005; Manning et al. 2011; McStay et al. 2014a; Weiss et al. 2015; Zaidman-Zait et al. 2017). However, three studies found no significant PS-EBP relationship whilst accounting for ASD severity (Bader et al. 2015; Falk et al. 2014; Firth and Dryer 2013). In these studies, child ASD severity *did* significantly account for variance in the DV. It therefore remains a possibility that the relationship between PS and EBP is to some extent accounted for by ASD severity. Regarding parent MHP, three studies found a significant relationship with EBP whilst accounting for ASD severity (which itself did not contribute to explained variance). Only Falk et al. (2014) report a significant association between MHP and ASD symptoms, whilst child EBP did not reach the criterion for entry into this stepwise regression.

#### Parent Factors

Parent age was entered in five studies, but was only significantly related to the dependent variable in that of Falk et al. (2014). Here, increasing maternal age was related to lower levels of maternal stress (*β* = − .24, *p* < .001) and anxiety (*β* = − .23, *p* < .001), whilst child EBP did not share unique variance with either of these dependent variables. This was a well-powered study, thus parental age cannot be ignored as a potential statistical mediator of the relationships of interest.

Parent use of coping mechanisms was investigated in three studies with PS as the DV (Manning et al. 2011; McStay et al. 2014b; Zaidman-Zait et al. 2017). Although coping mechanisms showed significant relationships with PS, child EBP retained a significant association, suggesting it has an independent effect. On the other hand, when parent perception of their own parenting was present in the models, the relationship of PS and parent MHP with child EBP became non-significant in three (Bader et al. 2015; Falk et al. 2014; Hastings and Brown 2002) out of four studies. Only in the largest study did both child EBP and parent perception of their own parenting both appear to contribute significantly to parent MHP (Suzuki et al. 2015). Parents’ perception of their ability to parent effectively is therefore a plausible candidate for a mediator of the relationship between child EBP and parent psychological distress variables.

#### Family and Contextual Factors

Perceived social and family support was commonly included in analyses and often made a significant contribution to variance in the DV. However, in most cases, the relationship of interest also remained significant. Cases in which the relationship did not remain significant tended to have a smaller sample size (e.g. Warfield et al. 2014; Gallagher et al. 2008). This suggests that social and family support may have an important relationship with parent MHP and PS; however, this relationship is independent of that with child EBP. Socio-economic status and family income rarely showed significant association with the dependent variables. Although some degree of relatedness to child EBP (Weiss et al. 2015), PS and parent MHP (Jones et al. 2014) was observed, the relationships between parent and child variables of interest remained significant in these cases.

### Longitudinal Analyses (Objective 4)

Baseline characteristics of the eleven included studies are provided in Table 1 and data regarding longitudinal analyses are given in Table 5. A variety of design and statistical approaches has been taken among these studies. Cross-time zero-order correlations (unadjusted association between a measure at an earlier and another measure at a later time-point) showed significant relationships between earlier PS and later child EBP in two studies of over 100 participants (Peters-Scheffer et al. 2012; Zaidman-Zait et al. 2014), but not in three studies with under 100 participants (Bader and Barry 2014; Reed et al. 2013; Simonoff et al. 2013). A similar pattern was shown for the association of earlier child EBP to later PS, and both directions of association between child EBP and parent MHP (Baker et al. 2011; Simonoff et al. 2013; Totsika et al. 2013).

**Table 5:** Longitudinal analyses

Study	Sample size (*n*)	*N* time points	Mean interval (months)	Retention (%)	Child scale (subscale)	Parent scale (subscale)	Type of analysis (statistic)	Child-to-parent prediction	Parent-to-child prediction	Other variables in model
Zaidman-Zait et al. (2014)	184	4	12	NR	CBCL 1.5-5 (ext.)	PSI-SF (PD)	Cross time correlation Cross-lagged SEM	* **	* n.s./**^c^	–
Baker et al. (2011)	149	2	36	89.2	SIB-R	CES-D	Cross time correlation Cross-lagged SEM	* n.s.	* n.s.	Mother–child relationship quality; family adaptability
Totsika et al. (2013)	132	2	24	NR	SDQ	K6	Cross-time correlation Cross-lagged SEM	n.s. n.s.	** *	Family deprivation
Benson (2014)	113	3^a^	24	84.0	NCBRF	Own MHP scale (change over time)	Multilevel modelling	*	-	Child age; child pro-social behavior; parent use of coping mechanisms
Peters-Scheffer et al. (2012)	104	3	12	NR	CBCL 1.5-5	Dutch PSI-SF	Cross time correlation Cross-lagged SEM	** n.s.	** n.s.	–
Bader et al. (2014)	84	2	25	75.7	CBCL (ext.)	PSI-SF (PD)	Cross time correlation	–	n.s.	–
Simonoff et al. (2013)	81	2	48	47.4	SDQ	GHQ-30 PSI-SF (PD)	Cross time correlation	–	n.s. n.s.	–
Osborne and Reed (2009) (2)	72	2	9.5	100	SDQ & DBC	PSI	ANCOVA	n.s.	*	Child ASD severity, cognitive ability; adaptive functioning
Osborne and Reed (2009) (1)	65	2	9.5	100	CPRS (oppos.)	QRS-F	ANCOVA	n.s.	**	Child ASD severity; cognitive ability; adaptive functioning
Hastings et al. (2014)	60	2	33	33	SDQ	HADS	MR controlling for DV T1 scores	–	n.s.	Sibling SDQ; sibling prosocial behavior
Lecavalier et al. (2006)	50	2	12	NR^b^	NCBRF	PSI-SF	HMR controlling for DV T1 scores	*	****	–
Reed et al. (2013)	32	2	9.5	100	DBC-P	QRS-F	Cross time correlation	–	†	–

*NR* not reported; *SEM* structural equation modelling; *ANCOVA* analysis of covariance; *MR* multiple regression; *HMR* hierarchical multiple regression; *DV* dependent variable

*n.s*. non-significant; ^†^*p* < .1; *p < .05; **p < .01; ***p < .001; ****p < .0001

^a^Accelerated longitudinal design

^b^Fifty (19.8%) out of 253 Time 1 participants provided data at Time 2, but it is unclear what proportion of the initial sample was eligible for/contacted at Time 2

^c^In the model, the path from Time 1 PS to Time 2 EBP was not significant; however the path from Time 3 PS to Time 4 EBP was significant (*p* < .01)

However, in analyses controlling for variable stability over time (i.e. the association of a DV with itself at an earlier time-point), associations tended to disappear (Baker et al. 2011; Hastings et al. 2014; Peters-Scheffer et al. 2012). Exceptions included the largest study (Zaidman-Zait et al. 2014), which found that earlier child EBP predicted later PS and vice versa (though only in the second time interval). In a smaller study, Lecavalier and colleagues (2006) found similar bidirectional associations, though the effect size was larger for the parent-to-child than the child-to-parent. Another large study (Totsika et al. 2013) found earlier parent MHP to predict later child EBP, but not vice versa.

Significant cross-time relationships have also been found whilst adjusting for other factors. Osborne and Reed (2009) found in two samples that earlier PS predicted later child EBP, whilst controlling for child characteristics (ASD severity, cognitive ability and adaptive functioning). The opposite (child-to-parent) predictive relationship was not evident. It may be that earlier child characteristics share their predictive relationship for PS, whereas earlier PS shows predictive ability for EBP that is separate from other child characteristics such as ASD severity.

Finally, Benson (2014) investigated moderation of the association between child EBP and change over time in parent MHP by parent use of a variety of coping mechanisms. He found an attenuated EBP-MHP relationship in parents who reported high use of a cognitive reframing strategy. This strategy involves actively changing ones perception of a stressor. Despite inclusion of this interaction term in the multilevel modelling analysis, the relationship between child EBP and change in parent MHP remained significant.

## Discussion

This review aimed systematically to collate the published evidence pertaining to the relationships between additional EBP in children with ASD and their parents’ MHP and PS. A series of meta-analyses showed the magnitude of unadjusted associations between these factors to be in the moderate range. The pooled estimates for the MHP-EBP analyses were slightly stronger than those found in comparable meta-analyses conducted by Goodman et al. (2011) in the general population. This remained true after controlling for inflation by small study effects (using the trim and fill procedure). Pooled correlation was lower in our analysis of effect sizes based on measures of child EBP rated by an alternative informant to the parent self-reporting on own MHP. However, when Goodman and colleagues performed comparable subset analyses, pooled estimates remained lower than our corresponding results for child EBP ratings by the mother, child themselves and by an observer. Goodman et al. did find a stronger pooled correlation ($\bar {r}=.34$) for studies in which mothers were recruited from clinical settings. Since mothers of children with ASD are already at increased risk for clinically relevant psychological distress (Jokiranta et al. 2013), our results and those of Goodman et al. appear to provide converging evidence that at higher levels of parental psychological distress, its relationship with child EBP is stronger. Although the current research was not equipped for further investigation of this possibility, our findings highlight the importance of investigating these associations in families of children with ASD. Consistent with Goodman and colleagues’ research, associations appeared similar for internalizing and externalizing child difficulties. Indicating the need to continue to investigate both of these factors in future research.

The PS-total EBP pooled association appeared greater in magnitude than that for MHP-total EBP; however, it also showed higher heterogeneity. We found some evidence that this heterogeneity may be driven at least in part by the similarity in content between child EBP questionnaires, and PS questionnaires that include ratings of child behavior. Several research groups have chosen to use subscales of PS questionnaires that minimize overlap between the concepts of child behavior problems themselves, and parent perception of the stress related to them (Bader et al. 2015; Simonoff et al. 2013; Zaidman-Zait et al. 2014). This approach may be advantageous in future research. Regarding MHP measures, there was no evidence that type of parent MHP measured (depression, anxiety or both) accounted for variation in effect size reported by different studies. It is worth acknowledging that parental MHP is not limited to mood disorders such as depression and anxiety and that other classes of mental health disorder (e.g. psychosis) could have different relationships with additional child EBP. However, these remain largely unexplored to date.

In the vast majority of studies, the informant for child EBP was the same parent that rated their own stress and MHP. This is potentially problematic in two ways. Firstly, correlations could be inflated by common method effects (Podsakoff et al. 2003), whereby questionnaire data collected from the same source may be related for reasons other than a true association between the concepts of interest. In this context specifically, it is possible that parents who experience more psychological distress also perceive the same child behaviors as more problematic than do other parents (Najman et al. 2000). Secondly, reliance on a single information source (mainly mothers) for child EBP may limit the applicability of the relationships seen with parent psychological distress to certain conditions. Elements of child behavior that parents find problematic may not align completely with elements that are problematic for others, including the children themselves. However, parent agreement with other informants appears to be moderate for the externalizing and internalizing behavior problems of children with ASD (Stratis and Lecavalier 2015). This suggests that parent-report captures at least some variance in common with that indexed by other reporters.

We found that in six studies where an effect size deriving from an alternative rater was available, the pooled estimate for the EBP-MHP association was lower, but remained significant. These results are similar to those found in the general population by Goodman et al. (2011), in which child EBP ratings by mothers yielded a larger pooled association with maternal depression than those yielded by observer or child self-report ratings (which were nevertheless significant). Together, these findings suggest that although the possibility of inflation by shared rater effects remains, these cannot account for the entire observed association. Using multiple informants may be the best way to home in on a more pervasive element of variance in child EBP (Stratis and Lecavalier 2015).

Our narrative review of adjusted concurrent relationships showed that in general, the associations between child EBP and parent MHP and PS are robust, as they tend to remain present after statistically accounting for other factors. This is especially true in larger studies. However, there is some evidence that this covariance may be shared with ASD severity and parent self-rated psychological factors such as coping mechanisms and perception of own parenting ability. This is supported by longitudinal research finding that the relationship between earlier parenting stress and later child EBP was mediated by parent self-rated ability to set limits for their child’s behavior (Osborne et al. 2008). (This study was not included in this review since it was based on the same sample as another study that used analyses more relevant to our aims.) This is consistent with a model proposed by Hastings (2002) in which child EBP increases parenting stress, which in turn compromises a parent’s ability to engage in positive parenting behaviors that would usually serve to support the child’s emotional and behavioral functioning. It is plausible that parent use of coping strategies to deal with stress may moderate the relationship between child EBP and PS, or between PS and parenting behavior. Evidence for the former was found by Benson (2014), as outlined in the longitudinal analyses section, above.

Although there was insufficient consistency across studies in other included variables to synthesize evidence regarding mediation and moderation, longitudinal studies provided some consistent evidence for the existence of bidirectional relationships between parent psychological well-being and child EBP over time. Studies with sample sizes of under 100 may be underpowered to detect associations, particularly whilst adjusting for other factors. Research finding bidirectional relationships in the general population has benefited from much larger samples (e.g. Bagner et al. 2013; Stone et al. 2016; Zadeh et al. 2010). This review therefore highlights the value of conducting further well-powered longitudinal research into the temporal relationship between child and parent psychological well-being in families with ASD. Establishing the structure of these relationships, and the additional factors that mediate and moderate them, may yield explanations as to the increased rate of MHP in both children with ASD and their parents, as well as guiding intervention research.

### Strengths

To our knowledge, this is the first systematic review of the growing literature on the relationships between additional EBP in children with ASD and psychological distress in their parents. It provides both a quantitative and qualitative synthesis of such research, yielding a comprehensive overview and basis for guiding future research. Strengths of the meta-analytic component include efforts to obtain additional data from study authors, and reliability checks for the screening and coding of studies. Disaggregation of child externalizing and internalizing problems allowed us to show that the relationships with parent MHP and PS observed for total EBP are likely not solely driven by either, but rather that both may be important factors. This is consistent with research in the general population (Goodman et al. 2011). Strengths of the qualitative syntheses include their focus on specific research questions aiming to guide future research.

### Limitations

A limitation of this review is its reliance on published research. Although there was little statistical evidence for publication bias in the parent MHP-total EBP analysis, significant evidence was present in the PS-total EBP analysis. Unfortunately, it was not possible to adequately disentangle the effects of potential publication bias from residual between-study heterogeneity. However, the number of unpublished null results required to render the PS-total EBP relationship non-significant was very large. Therefore, whilst it remains a possibility that the pooled effect size is inflated due to publication bias, it is unlikely that publication bias can completely explain the observed association.

A further limitation of this review is that it did not consider measurement of PS and MHP, other than by parent self-report. Several recent studies have used cortisol as a biomarker for stress in parents of children with ASD (e.g. Bitsika et al. 2017; Dykens and Lambert 2013). Although not reviewed here, these studies contribute importantly to the literature on PS in ASD, since stress by definition includes a physiological component related to both physical and mental health outcomes (Dykens and Lambert 2013).

## Conclusions

Systematic synthesis of research into the relationship between additional MHP in children with ASD and stress levels and mental health of their parents has overall shown robust relationships both concurrently and across time. Future research should focus on further investigating the additional factors involved in these relationships in an organized and hypothesis-driven manner, based on evidence from existing research. Progression in this essential area of research should come from well-powered longitudinal studies using well-defined measures from a variety of information sources.

## Electronic supplementary material

Below is the link to the electronic supplementary material.

Supplementary material 1 (PDF 154 KB)
