# Correction to: The Association Between Emotional and Behavioral Problems in Children with Autism Spectrum Disorder and Psychological Distress in Their Parents: A Systematic Review and Meta-analysis

**DOI:** 10.1007/s10803-018-3656-0

**Published:** 2018-07-18

**Authors:** Isabel Yorke, Pippa White, Amelia Weston, Monica Rafla, Tony Charman, Emily Simonoff

**Affiliations:** 1 Institute of Psychiatry, Psychology & Neuroscience, King’s College London, 16 De Crespigny Park, London, SE5 8AF UK; 2 Department of Psychology, University of Bath, Bath, UK

## Correction to: Journal of Autism and Developmental Disorders 10.1007/s10803-018-3605-y

The original version of this article contains additional supplementary material which was omitted during the initial submission and it was not published. These are graphical representations of the studies and effect sizes in each meta-analysis. Please note that there is no change to the main body of the article, data entered, or conclusions drawn from the meta-analyses.

## Electronic supplementary material

Below is the link to the electronic supplementary material.

Supplementary material 1 (DOCX 306 KB)

