# Supplementary material for: The Association Between Emotional and Behavioral Problems in Children with Autism Spectrum Disorder and Psychological Distress in Their Parents: A Systematic Review and Meta-analysis
Source: J Autism Dev Disord. 2018 May 18;48(10):3393–415. doi: 10.1007/s10803-018-3605-y (PMC6153902; doi:10.1007/s10803-018-3605-y)
Supplement: Supplementary file 1 — Supplementary material 1 (PDF 154 KB) [file 10803_2018_3605_MOESM1_ESM.pdf]

Supplemental Material for: The association between emotional and behavioural problems in children with ASD and stress and mental health problems in their parents: a systematic review and meta-analysis.

Journal of Autism and Developmental Disorders

Isabel Yorke[1], Pippa White[1], Amelia Weston[2], Monica Rafla[1], Tony Charman[1], Emily Simonoff[1]

[1]King's College London, Institute of Psychiatry, Psychology & Neuroscience, London, UK

[2]University of Bath, Department of Psychology, Bath, UK

Correspondence: Isabel Yorke, Institute of Psychiatry, Psychology & Neuroscience, 16 De Crespigny Park, London, SE5 8AF; 0207 848 5832; isabel.yorke@kcl.ac.uk.

orcid.org/0000-0001-8603-0910

Supplemental Material 1. Exact search strategies run in each database

OVID Medline

1. exp Child Development Disorders, Pervasive/

2. autis*.mp. [mp=title, abstract, original title, name of substance word, subject heading word, keyword heading word, protocol supplementary concept word, rare disease supplementary concept word, unique identifier, synonyms]

3. asperger*.mp. [mp=title, abstract, original title, name of substance word, subject heading word, keyword heading word, protocol supplementary concept word, rare disease supplementary concept word, unique identifier, synonyms]

4. (ASD not ("atrial septal defect" or "acute stress disorder")).mp. [mp=title, abstract, original title, name of substance word, subject heading word, keyword heading word, protocol supplementary concept word, rare disease supplementary concept word, unique identifier, synonyms]

5. pervasive developmental disorder.mp. [mp=title, abstract, original title, name of substance word, subject heading word, keyword heading word, protocol supplementary concept word, rare disease supplementary concept word, unique identifier, synonyms]

6. exp anxiety disorders/ or exp "disruptive, impulse control, and conduct disorders"/ or exp mood disorders/ or exp anxiety, separation/ or exp "attention deficit and disruptive behavior disorders"/ or exp child behavior disorders/

7. maladaptive.mp. [mp=title, abstract, original title, name of substance word, subject heading word, keyword heading word, protocol supplementary concept word, rare disease supplementary concept word, unique identifier, synonyms]

8. challenging behavio*.mp. [mp=title, abstract, original title, name of substance word, subject heading word, keyword heading word, protocol supplementary concept word, rare disease supplementary concept word, unique identifier, synonyms]

9. self-injur*.mp. [mp=title, abstract, original title, name of substance word, subject heading word, keyword heading word, protocol supplementary concept word, rare disease supplementary concept word, unique identifier, synonyms]

10. self-harm.mp. [mp=title, abstract, original title, name of substance word, subject heading word, keyword heading word, protocol supplementary concept word, rare disease supplementary concept word, unique identifier, synonyms]

11. mood.mp. [mp=title, abstract, original title, name of substance word, subject heading word, keyword heading word, protocol supplementary concept word, rare disease supplementary concept word, unique identifier, synonyms]

12. (aggress* not cancer).mp. [mp=title, abstract, original title, name of substance word, subject heading word, keyword heading word, protocol supplementary concept word, rare disease supplementary concept word, unique identifier, synonyms]

13. conduct disorder.mp. [mp=title, abstract, original title, name of substance word, subject heading word, keyword heading word, protocol supplementary concept word, rare disease supplementary concept word, unique identifier, synonyms]

14. conduct problem*.mp. [mp=title, abstract, original title, name of substance word, subject heading word, keyword heading word, protocol supplementary concept word, rare disease supplementary concept word, unique identifier, synonyms]

15. anxi*.mp. [mp=title, abstract, original title, name of substance word, subject heading word, keyword heading word, protocol supplementary concept word, rare disease supplementary concept word, unique identifier, synonyms]

16. depress*.mp. [mp=title, abstract, original title, name of substance word, subject heading word, keyword heading word, protocol supplementary concept word, rare disease supplementary concept word, unique identifier, synonyms]

17. ADHD.mp. [mp=title, abstract, original title, name of substance word, subject heading word, keyword heading word, protocol supplementary concept word, rare disease supplementary concept word, unique identifier, synonyms]

18. attention-deficit.mp. [mp=title, abstract, original title, name of substance word, subject heading word, keyword heading word, protocol supplementary concept word, rare disease supplementary concept word, unique identifier, synonyms]

19. oppositional defiant.mp. [mp=title, abstract, original title, name of substance word, subject heading word, keyword heading word, protocol supplementary concept word, rare disease supplementary concept word, unique identifier, synonyms]

20. (internalising or internalizing).mp. [mp=title, abstract, original title, name of substance word, subject heading word, keyword heading word, protocol supplementary concept word, rare disease supplementary concept word, unique identifier, synonyms]

21. (externalising or externalizing).mp. [mp=title, abstract, original title, name of substance word, subject heading word, keyword heading word, protocol supplementary concept word, rare disease supplementary concept word, unique identifier, synonyms]

22. problem* behavio*.mp. [mp=title, abstract, original title, name of substance word, subject heading word, keyword heading word, protocol supplementary concept word, rare disease supplementary concept word, unique identifier, synonyms]

23. behavio* problem*.mp. [mp=title, abstract, original title, name of substance word, subject heading word, keyword heading word, protocol supplementary concept word, rare disease supplementary concept word, unique identifier, synonyms]

24. mental health.mp. [mp=title, abstract, original title, name of substance word, subject heading word, keyword heading word, protocol supplementary concept word, rare disease supplementary concept word, unique identifier, synonyms]

25. mental disorder*.mp. [mp=title, abstract, original title, name of substance word, subject heading word, keyword heading word, protocol supplementary concept word, rare disease supplementary concept word, unique identifier, synonyms]

26. psychiatr*.mp. [mp=title, abstract, original title, name of substance word, subject heading word, keyword heading word, protocol supplementary concept word, rare disease supplementary concept word, unique identifier, synonyms]

27. psychopatholog*.mp. [mp=title, abstract, original title, name of substance word, subject heading word, keyword heading word, protocol supplementary concept word, rare disease supplementary concept word, unique identifier, synonyms]

28. comorbid*.mp. [mp=title, abstract, original title, name of substance word, subject heading word, keyword heading word, protocol supplementary concept word, rare disease supplementary concept word, unique identifier, synonyms]

29. exp family characteristics/ or exp family relations/

30. ((stress* or distress* or psychopatholog* or psychiatr* or mental health or mental disorder* or behavio* or warmth or authorit* or overcontrol or control or self-efficacy or confidence or engagement) adj2 (parent* or mother* or father* or maternal or paternal or caregiver*)).mp.

31. (son adj1 father).mp. [mp=title, abstract, original title, name of substance word, subject heading word, keyword heading word, protocol supplementary concept word, rare disease supplementary concept word, unique identifier, synonyms]

32. (daughter adj1 father).mp. [mp=title, abstract, original title, name of substance word, subject heading word, keyword heading word, protocol supplementary concept word, rare disease supplementary concept word, unique identifier, synonyms]

33. (child adj1 father).mp. [mp=title, abstract, original title, name of substance word, subject heading word, keyword heading word, protocol supplementary concept word, rare disease supplementary concept word, unique identifier, synonyms]

34. (son adj1 mother).mp. [mp=title, abstract, original title, name of substance word, subject heading word, keyword heading word, protocol supplementary concept word, rare disease supplementary concept word, unique identifier, synonyms]

35. (daughter adj1 mother).mp. [mp=title, abstract, original title, name of substance word, subject heading word, keyword heading word, protocol supplementary concept word, rare disease supplementary concept word, unique identifier, synonyms]

36. (child adj1 mother).mp. [mp=title, abstract, original title, name of substance word, subject heading word, keyword heading word, protocol supplementary concept word, rare disease supplementary concept word, unique identifier, synonyms]

37. (son adj1 parent).mp. [mp=title, abstract, original title, name of substance word, subject heading word, keyword heading word, protocol supplementary concept word, rare disease supplementary concept word, unique identifier, synonyms]

38. (daughter adj1 parent).mp. [mp=title, abstract, original title, name of substance word, subject heading word, keyword heading word, protocol supplementary concept word, rare disease supplementary concept word, unique identifier, synonyms]

39. (child adj1 parent).mp. [mp=title, abstract, original title, name of substance word, subject heading word, keyword heading word, protocol supplementary concept word, rare disease supplementary concept word, unique identifier, synonyms]

40. (son adj1 caregiver).mp. [mp=title, abstract, original title, name of substance word, subject heading word, keyword heading word, protocol supplementary concept word, rare disease supplementary concept word, unique identifier, synonyms]

41. (daughter adj1 caregiver).mp. [mp=title, abstract, original title, name of substance word, subject heading word, keyword heading word, protocol supplementary concept word, rare disease supplementary concept word, unique identifier, synonyms]

42. (child adj1 caregiver).mp. [mp=title, abstract, original title, name of substance word, subject heading word, keyword heading word, protocol supplementary concept word, rare disease supplementary concept word, unique identifier, synonyms]

43. 31 or 32 or 33 or 34 or 35 or 36 or 37 or 38 or 39 or 40 or 41 or 42

44. 1 or 2 or 3 or 4 or 5

45. 6 or 7 or 8 or 9 or 10 or 11 or 12 or 13 or 14 or 15 or 16 or 17 or 18 or 19 or 20 or 21 or 22 or 23 or 24 or 25 or 26 or 27 or 28

46. 29 or 30 or 43

47. 44 and 45 and 46

48. limit 47 to (english language and yr="2000 -Current")

PsycARTICLES

1. autis*.mp. [mp=title, abstract, full text, caption text]

2. asperger*.mp. [mp=title, abstract, full text, caption text]

3. (ASD not ("atrial septal defect" or "acute stress disorder")).mp. [mp=title, abstract, full text, caption text]

4. pervasive developmental disorder.mp. [mp=title, abstract, full text, caption text]

5. maladaptive.mp. [mp=title, abstract, full text, caption text]

6. challenging behavio*.mp. [mp=title, abstract, full text, caption text]

7. self-injur*.mp. [mp=title, abstract, full text, caption text]

8. self-harm.mp. [mp=title, abstract, full text, caption text]

9. mood.mp. [mp=title, abstract, full text, caption text]

10. (aggress* not cancer).mp. [mp=title, abstract, full text, caption text]

11. conduct disorder.mp. [mp=title, abstract, full text, caption text]

12. conduct problem*.mp. [mp=title, abstract, full text, caption text]

13. anxi*.mp. [mp=title, abstract, full text, caption text]

14. depress*.mp. [mp=title, abstract, full text, caption text]

15. ADHD.mp. [mp=title, abstract, full text, caption text]

16. attention-deficit.mp. [mp=title, abstract, full text, caption text]

17. oppositional defiant.mp. [mp=title, abstract, full text, caption text]

18. (internalising or internalizing).mp. [mp=title, abstract, full text, caption text]

19. (externalising or externalizing).mp. [mp=title, abstract, full text, caption text]

20. problem* behavio*.mp. [mp=title, abstract, full text, caption text]

21. behavio* problem*.mp. [mp=title, abstract, full text, caption text]

22. mental health.mp. [mp=title, abstract, full text, caption text]

23. mental disorder*.mp. [mp=title, abstract, full text, caption text]

24. psychiatr*.mp. [mp=title, abstract, full text, caption text]

25. psychopatholog*.mp. [mp=title, abstract, full text, caption text]

26. comorbid*.mp. [mp=title, abstract, full text, caption text]

27. ((stress* or distress* or psychopatholog* or psychiatr* or mental health or mental disorder* or behavio* or warmth or authorit* or overcontrol or control or self-efficacy or confidence or engagement) adj2 (parent* or mother* or father* or maternal or paternal or caregiver*)).mp.

28. (son adj1 father).mp. [mp=title, abstract, full text, caption text]

29. (daughter adj1 father).mp. [mp=title, abstract, full text, caption text]

30. (child adj1 father).mp. [mp=title, abstract, full text, caption text]

31. (son adj1 mother).mp. [mp=title, abstract, full text, caption text]

32. (daughter adj1 mother).mp. [mp=title, abstract, full text, caption text]

33. (child adj1 mother).mp. [mp=title, abstract, full text, caption text]

34. (son adj1 parent).mp. [mp=title, abstract, full text, caption text]

35. (daughter adj1 parent).mp. [mp=title, abstract, full text, caption text]

36. (child adj1 parent).mp. [mp=title, abstract, full text, caption text]

37. (son adj1 caregiver).mp. [mp=title, abstract, full text, caption text]

38. (daughter adj1 caregiver).mp. [mp=title, abstract, full text, caption text]

39. (child adj1 caregiver).mp. [mp=title, abstract, full text, caption text]

40. 28 or 29 or 30 or 31 or 32 or 33 or 34 or 35 or 36 or 37 or 38 or 39

41. 5 or 6 or 7 or 8 or 9 or 10 or 11 or 12 or 13 or 14 or 15 or 16 or 17 or 18 or 19 or 20 or 21 or 22 or 23 or 24 or 25 or 26

42. 27 or 40

43. 1 or 2 or 3 or 4

44. 41 and 42 and 43

45. limit 44 to yr="2000 -Current"

Embase

1. exp Child Development Disorders, Pervasive/

2. autis*.mp. [mp=title, abstract, heading word, drug trade name, original title, device manufacturer, drug manufacturer, device trade name, keyword, floating subheading]

3. asperger*.mp. [mp=title, abstract, heading word, drug trade name, original title, device manufacturer, drug manufacturer, device trade name, keyword, floating subheading]

4. (ASD not ("atrial septal defect" or "acute stress disorder")).mp. [mp=title, abstract, heading word, drug trade name, original title, device manufacturer, drug manufacturer, device trade name, keyword, floating subheading]

5. pervasive developmental disorder.mp. [mp=title, abstract, heading word, drug trade name, original title, device manufacturer, drug manufacturer, device trade name, keyword, floating subheading]

6. exp anxiety disorders/ or exp "disruptive, impulse control, and conduct disorders"/ or exp mood disorders/ or exp anxiety, separation/ or exp "attention deficit and disruptive behavior disorders"/ or exp child behavior disorders/

7. maladaptive.mp. [mp=title, abstract, heading word, drug trade name, original title, device manufacturer, drug manufacturer, device trade name, keyword, floating subheading]

8. challenging behavio*.mp. [mp=title, abstract, heading word, drug trade name, original title, device manufacturer, drug manufacturer, device trade name, keyword, floating subheading]

9. self-injur*.mp. [mp=title, abstract, heading word, drug trade name, original title, device manufacturer, drug manufacturer, device trade name, keyword, floating subheading]

10. self-harm.mp. [mp=title, abstract, heading word, drug trade name, original title, device manufacturer, drug manufacturer, device trade name, keyword, floating subheading]

11. mood.mp. [mp=title, abstract, heading word, drug trade name, original title, device manufacturer, drug manufacturer, device trade name, keyword, floating subheading]

12. (aggress* not cancer).mp. [mp=title, abstract, heading word, drug trade name, original title, device manufacturer, drug manufacturer, device trade name, keyword, floating subheading]

13. conduct disorder.mp. [mp=title, abstract, heading word, drug trade name, original title, device manufacturer, drug manufacturer, device trade name, keyword, floating subheading]

14. conduct problem*.mp. [mp=title, abstract, heading word, drug trade name, original title, device manufacturer, drug manufacturer, device trade name, keyword, floating subheading]

15. anxi*.mp. [mp=title, abstract, heading word, drug trade name, original title, device manufacturer, drug manufacturer, device trade name, keyword, floating subheading]

16. depress*.mp. [mp=title, abstract, heading word, drug trade name, original title, device manufacturer, drug manufacturer, device trade name, keyword, floating subheading]

17. ADHD.mp. [mp=title, abstract, heading word, drug trade name, original title, device manufacturer, drug manufacturer, device trade name, keyword, floating subheading]

18. attention-deficit.mp. [mp=title, abstract, heading word, drug trade name, original title, device manufacturer, drug manufacturer, device trade name, keyword, floating subheading]

19. oppositional defiant.mp. [mp=title, abstract, heading word, drug trade name, original title, device manufacturer, drug manufacturer, device trade name, keyword, floating subheading]

20. (internalising or internalizing).mp. [mp=title, abstract, heading word, drug trade name, original title, device manufacturer, drug manufacturer, device trade name, keyword, floating subheading]

21. (externalising or externalizing).mp. [mp=title, abstract, heading word, drug trade name, original title, device manufacturer, drug manufacturer, device trade name, keyword, floating subheading]

22. problem* behavio*.mp. [mp=title, abstract, heading word, drug trade name, original title, device manufacturer, drug manufacturer, device trade name, keyword, floating subheading]

23. behavio* problem*.mp. [mp=title, abstract, heading word, drug trade name, original title, device manufacturer, drug manufacturer, device trade name, keyword, floating subheading]

24. mental health.mp. [mp=title, abstract, heading word, drug trade name, original title, device manufacturer, drug manufacturer, device trade name, keyword, floating subheading]

25. mental disorder*.mp. [mp=title, abstract, heading word, drug trade name, original title, device manufacturer, drug manufacturer, device trade name, keyword, floating subheading]

26. psychiatr*.mp. [mp=title, abstract, heading word, drug trade name, original title, device manufacturer, drug manufacturer, device trade name, keyword, floating subheading]

27. psychopatholog*.mp. [mp=title, abstract, heading word, drug trade name, original title, device manufacturer, drug manufacturer, device trade name, keyword, floating subheading]

28. comorbid*.mp. [mp=title, abstract, heading word, drug trade name, original title, device manufacturer, drug manufacturer, device trade name, keyword, floating subheading]

29. exp family characteristics/ or exp family relations/

30. ((stress* or distress* or psychopatholog* or psychiatr* or mental health or mental disorder* or behavio* or warmth or authorit* or overcontrol or control or self-efficacy or confidence or engagement) adj2 (parent* or mother* or father* or maternal or paternal or caregiver*)).mp.

31. (son adj1 father).mp. [mp=title, abstract, heading word, drug trade name, original title, device manufacturer, drug manufacturer, device trade name, keyword, floating subheading]

32. (daughter adj1 father).mp. [mp=title, abstract, heading word, drug trade name, original title, device manufacturer, drug manufacturer, device trade name, keyword, floating subheading]

33. (child adj1 father).mp. [mp=title, abstract, heading word, drug trade name, original title, device manufacturer, drug manufacturer, device trade name, keyword, floating subheading]

34. (son adj1 mother).mp. [mp=title, abstract, heading word, drug trade name, original title, device manufacturer, drug manufacturer, device trade name, keyword, floating subheading]

35. (daughter adj1 mother).mp. [mp=title, abstract, heading word, drug trade name, original title, device manufacturer, drug manufacturer, device trade name, keyword, floating subheading]

36. (child adj1 mother).mp. [mp=title, abstract, heading word, drug trade name, original title, device manufacturer, drug manufacturer, device trade name, keyword, floating subheading]

37. (son adj1 parent).mp. [mp=title, abstract, heading word, drug trade name, original title, device manufacturer, drug manufacturer, device trade name, keyword, floating subheading]

38. (daughter adj1 parent).mp. [mp=title, abstract, heading word, drug trade name, original title, device manufacturer, drug manufacturer, device trade name, keyword, floating subheading]

39. (child adj1 parent).mp. [mp=title, abstract, heading word, drug trade name, original title, device manufacturer, drug manufacturer, device trade name, keyword, floating subheading]

40. (son adj1 caregiver).mp. [mp=title, abstract, heading word, drug trade name, original title, device manufacturer, drug manufacturer, device trade name, keyword, floating subheading]

41. (daughter adj1 caregiver).mp. [mp=title, abstract, heading word, drug trade name, original title, device manufacturer, drug manufacturer, device trade name, keyword, floating subheading]

42. (child adj1 caregiver).mp. [mp=title, abstract, heading word, drug trade name, original title, device manufacturer, drug manufacturer, device trade name, keyword, floating subheading]

43. 31 or 32 or 33 or 34 or 35 or 36 or 37 or 38 or 39 or 40 or 41 or 42

44. 1 or 2 or 3 or 4 or 5

45. 6 or 7 or 8 or 9 or 10 or 11 or 12 or 13 or 14 or 15 or 16 or 17 or 18 or 19 or 20 or 21 or 22 or 23 or 24 or 25 or 26 or 27 or 28

46. 29 or 30 or 43

47. 44 and 45 and 46

48. limit 47 to (english language and yr="2000 -Current")

PsycINFO

1. autis*.mp. [mp=title, abstract, heading word, table of contents, key concepts, original title, tests & measures]

2. asperger*.mp. [mp=title, abstract, heading word, table of contents, key concepts, original title, tests & measures]

3. (ASD not ("atrial septal defect" or "acute stress disorder")).mp. [mp=title, abstract, heading word, table of contents, key concepts, original title, tests & measures]

4. pervasive developmental disorder.mp. [mp=title, abstract, heading word, table of contents, key concepts, original title, tests & measures]

5. maladaptive.mp. [mp=title, abstract, heading word, table of contents, key concepts, original title, tests & measures]

6. challenging behavio*.mp. [mp=title, abstract, heading word, table of contents, key concepts, original title, tests & measures]

7. self-injur*.mp. [mp=title, abstract, heading word, table of contents, key concepts, original title, tests & measures]

8. self-harm.mp. [mp=title, abstract, heading word, table of contents, key concepts, original title, tests & measures]

9. mood.mp. [mp=title, abstract, heading word, table of contents, key concepts, original title, tests & measures]

10. (aggress* not cancer).mp. [mp=title, abstract, heading word, table of contents, key concepts, original title, tests & measures]

11. conduct disorder.mp. [mp=title, abstract, heading word, table of contents, key concepts, original title, tests & measures]

12. conduct problem*.mp. [mp=title, abstract, heading word, table of contents, key concepts, original title, tests & measures]

13. anxi*.mp. [mp=title, abstract, heading word, table of contents, key concepts, original title, tests & measures]

14. depress*.mp. [mp=title, abstract, heading word, table of contents, key concepts, original title, tests & measures]

15. ADHD.mp. [mp=title, abstract, heading word, table of contents, key concepts, original title, tests & measures]

16. attention-deficit.mp. [mp=title, abstract, heading word, table of contents, key concepts, original title, tests & measures]

17. oppositional defiant.mp. [mp=title, abstract, heading word, table of contents, key concepts, original title, tests & measures]

18. (internalising or internalizing).mp. [mp=title, abstract, heading word, table of contents, key concepts, original title, tests & measures]

19. (externalising or externalizing).mp. [mp=title, abstract, heading word, table of contents, key concepts, original title, tests & measures]

20. problem* behavio*.mp. [mp=title, abstract, heading word, table of contents, key concepts, original title, tests & measures]

21. behavio* problem*.mp. [mp=title, abstract, heading word, table of contents, key concepts, original title, tests & measures]

22. mental health.mp. [mp=title, abstract, heading word, table of contents, key concepts, original title, tests & measures]

23. mental disorder*.mp. [mp=title, abstract, heading word, table of contents, key concepts, original title, tests & measures]

24. psychiatr*.mp. [mp=title, abstract, heading word, table of contents, key concepts, original title, tests & measures]

25. psychopatholog*.mp. [mp=title, abstract, heading word, table of contents, key concepts, original title, tests & measures]

26. comorbid*.mp. [mp=title, abstract, heading word, table of contents, key concepts, original title, tests & measures]

27. ((stress* or distress* or psychopatholog* or psychiatr* or mental health or mental disorder* or behavio* or warmth or authorit* or overcontrol or control or self-efficacy or confidence or engagement) adj2 (parent* or mother* or father* or maternal or paternal or caregiver*)).mp.

28. (son adj1 father).mp. [mp=title, abstract, heading word, table of contents, key concepts, original title, tests & measures]

29. (daughter adj1 father).mp. [mp=title, abstract, heading word, table of contents, key concepts, original title, tests & measures]

30. (child adj1 father).mp. [mp=title, abstract, heading word, table of contents, key concepts, original title, tests & measures]

31. (son adj1 mother).mp. [mp=title, abstract, heading word, table of contents, key concepts, original title, tests & measures]

32. (daughter adj1 mother).mp. [mp=title, abstract, heading word, table of contents, key concepts, original title, tests & measures]

33. (child adj1 mother).mp. [mp=title, abstract, heading word, table of contents, key concepts, original title, tests & measures]

34. (son adj1 parent).mp. [mp=title, abstract, heading word, table of contents, key concepts, original title, tests & measures]

35. (daughter adj1 parent).mp. [mp=title, abstract, heading word, table of contents, key concepts, original title, tests & measures]

36. (child adj1 parent).mp. [mp=title, abstract, heading word, table of contents, key concepts, original title, tests & measures]

37. (son adj1 caregiver).mp. [mp=title, abstract, heading word, table of contents, key concepts, original title, tests & measures]

38. (daughter adj1 caregiver).mp. [mp=title, abstract, heading word, table of contents, key concepts, original title, tests & measures]

39. (child adj1 caregiver).mp. [mp=title, abstract, heading word, table of contents, key concepts, original title, tests & measures]

40. 28 or 29 or 30 or 31 or 32 or 33 or 34 or 35 or 36 or 37 or 38 or 39

41. exp autism spectrum disorders/

42. exp anxiety disorders/ or exp impulse control disorders/ or exp attention deficit disorder/ or exp attention deficit disorder with hyperactivity/ or exp behavior disorders/ or exp conduct disorder/ or exp emotional disturbances/

43. exp parental characteristics/ or exp parenting/

44. 1 or 2 or 3 or 4 or 41

45. 5 or 6 or 7 or 8 or 9 or 10 or 11 or 12 or 13 or 14 or 15 or 16 or 17 or 18 or 19 or 20 or 21 or 22 or 23 or 24 or 25 or 26

46. 42 or 45

47. 44 and 46

48. 27 or 40 or 43

49. 47 and 48

50. limit 49 to (english language and yr="2000 -Current")

Cochrane

#1 "pervasive developmental disorder"

#2 autis*

#3 ASD not ("atrial septic defect" or "acute stress disorder")

#4 Asperger*

#5 MeSH descriptor: [Child Development Disorders, Pervasive] explode all trees

#6 #1 or #2 or #3 or #4 or #5

#7 maladaptive

#8 "challenging behavio*"

#9 self-injur* or self-harm

#10 mood

#11 aggress* not cancer

#12 "conduct problem*"

#13 "conduct disorder"

#14 anxi*

#15 depress*

#16 ADHD

#17 attention-deficit

#18 "oppositional defiant"

#19 internalising or internalizing

#20 externalizing or externalising

#21 "problem* behavio*"

#22 "behavio* problem*"

#23 "mental health"

#24 "mental disorder*"

#25 psychiatr*

#26 psychopatholog*

#27 comorbid*

#28 MeSH descriptor: [Disruptive, Impulse Control, and Conduct Disorders] 1 tree(s) exploded

#29 MeSH descriptor: [Anxiety Disorders] this term only

#30 MeSH descriptor: [Mood Disorders] explode all trees

#31 MeSH descriptor: [Anxiety, Separation] explode all trees

#32 MeSH descriptor: [Child Behavior Disorders] explode all trees

#33 MeSH descriptor: [Attention Deficit and Disruptive Behavior Disorders] explode all trees

#34 {or #7-#33}

#35 stress*

#36 distress*

#37 psychopatholog*

#38 psychiatr*

#39 "mental health"

#40 "mental disorder"

#41 warmth

#42 authorit*

#43 overcontrol

#44 control

#45 self-efficacy

#46 confidence

#47 engagement

#48 behavio*

#49 {or #35-#48}

#50 parent*

#51 mother*

#52 father*

#53 paternal

#54 maternal

#55 caregiver

#56 {or #50-#55}

#57 #49 near/2 #56

#58 son or daughter or child

#59 mother or father or parent or caregiver

#60 #58 near/1 #59

#61 MeSH descriptor: [Family Relations] explode all trees

#62 MeSH descriptor: [Family Characteristics] explode all trees

#63 #57 or #60 or #61 or #62

#64 #63 and #34 and #6

Web of Science Core Collection

#31 #30 AND #29 AND #28
DocType=All document types; Language=All languages;
#30 #27 OR #26
DocType=All document types; Language=All languages;
#29 #25 OR #24 OR #23 OR #22 OR #21 OR #20 OR #19 OR #18 OR #17 OR #16 OR #15 OR #14 OR #13 OR #12 OR #11 OR #10 OR #9 OR #8 OR #7 OR #6 OR #5
DocType=All document types; Language=All languages;
#28 #4 OR #3 OR #2 OR #1
DocType=All document types; Language=All languages;
#27 **TOPIC:** ((son or daughter or child) near/1 (father or mother or parent or caregiver))
DocType=All document types; Language=All languages;
#26 TS=((parent* OR mother* or father* or maternal or paternal or caregiver*) NEAR/2 (stress* or distress* or psychopatholog* or psychiatr* or "mental health" or "mental disorder*" or behavio* or warmth or authorit* or overcontrol or control or self-efficacy or confidence or engagement))
DocType=All document types; Language=All languages;
#25 **TOPIC:** (comorbid*)
DocType=All document types; Language=All languages;
#24 **TOPIC:** (psychopatholog*)
DocType=All document types; Language=All languages;
#23 **TOPIC:** (psychiatr*)
DocType=All document types; Language=All languages;
#22 **TOPIC:** ("mental disorder*")
DocType=All document types; Language=All languages;
#21 **TOPIC:** ("mental health")
DocType=All document types; Language=All languages;
#20 **TOPIC:** (problem* near/1 behavio*)
DocType=All document types; Language=All languages;
#19 **TOPIC:** (externali*ing)
DocType=All document types; Language=All languages;
#18 **TOPIC:** (internali*ing)
DocType=All document types; Language=All languages;
#17 **TOPIC:** ("oppositional defiant")
DocType=All document types; Language=All languages;
#16 **TOPIC:** (attention-deficit)
DocType=All document types; Language=All languages;
#15 **TOPIC:** (ADHD)
DocType=All document types; Language=All languages;
#14 **TOPIC:** (depress*)
DocType=All document types; Language=All languages;
#13 **TOPIC:** (anxi*)
DocType=All document types; Language=All languages;
#12 **TOPIC:** ("conduct problem*")
DocType=All document types; Language=All languages;

#11 **TOPIC:** ("conduct disorder")
DocType=All document types; Language=All languages;
#10 **TOPIC:** (aggress* NOT cancer)
DocType=All document types; Language=All languages;
#9 **TOPIC:** (mood)
DocType=All document types; Language=All languages;
#8 **TOPIC:** (self-injur*)
DocType=All document types; Language=All languages;
#7 **TOPIC:** (self-harm)
DocType=All document types; Language=All languages;
#6 **TOPIC:** ("challenging behavio*")
DocType=All document types; Language=All languages;
#5 **TOPIC:** (maladaptive)
DocType=All document types; Language=All languages;
#4 **TOPIC:** ("pervasive developmental disorder")
DocType=All document types; Language=All languages;
#3 TS=(ASD NOT ("atrial septal defect" or "acute distress disorder"))
DocType=All document types; Language=All languages;
#2 **TOPIC:** (Asperger*)
DocType=All document types; Language=All languages;
#1 **TOPIC:** (Autis*)
DocType=All document types; Language=All languages;
