# Supplementary material for: Correction to: The Association Between Emotional and Behavioral Problems in Children with Autism Spectrum Disorder and Psychological Distress in Their Parents: A Systematic Review and Meta-analysis
Source: J Autism Dev Disord. 2018 Jul 18;48(10):3416. doi: 10.1007/s10803-018-3656-0 (PMC6153899; doi:10.1007/s10803-018-3656-0)
Supplement: Supplementary file 1 — Supplementary material 1 (DOCX 306 KB) [file 10803_2018_3656_MOESM1_ESM.docx]

Additional Supplemental Material for: The association between emotional and behavioural problems in children with ASD and stress and mental health problems in their parents: a systematic review and meta-analysis.

Journal of Autism and Developmental Disorders.

Supplemental Material 2. Forest plots for all analyses


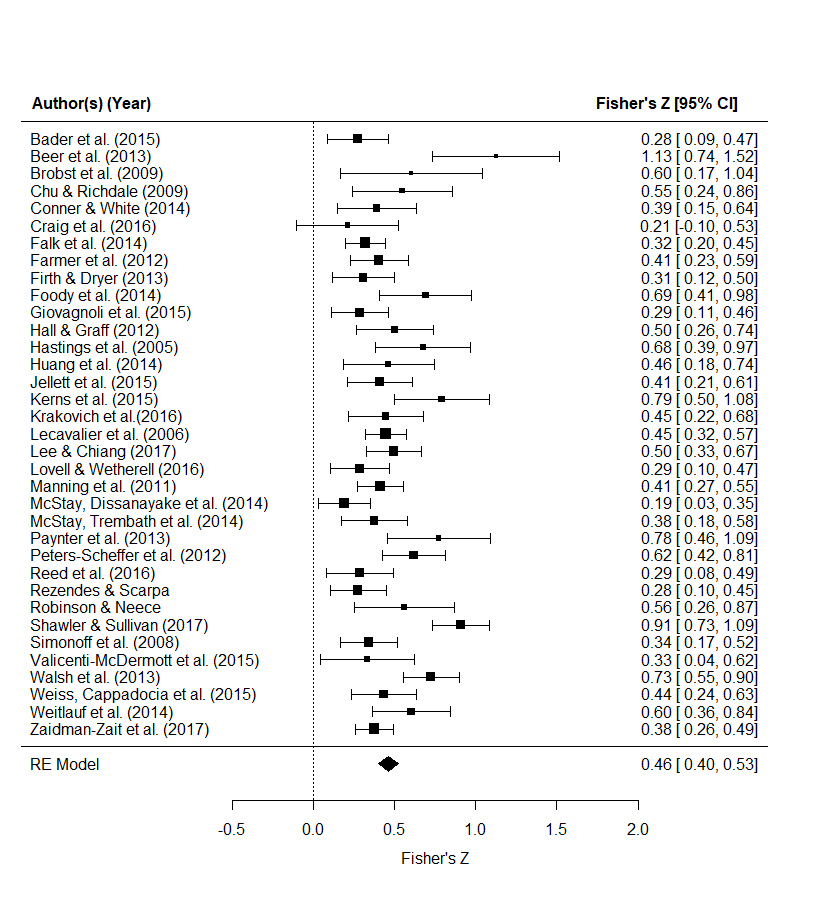


Supplementary Figure 1. Forest plot for Analysis 1 (associations between parenting stress and child total emotional and behavioral problems)


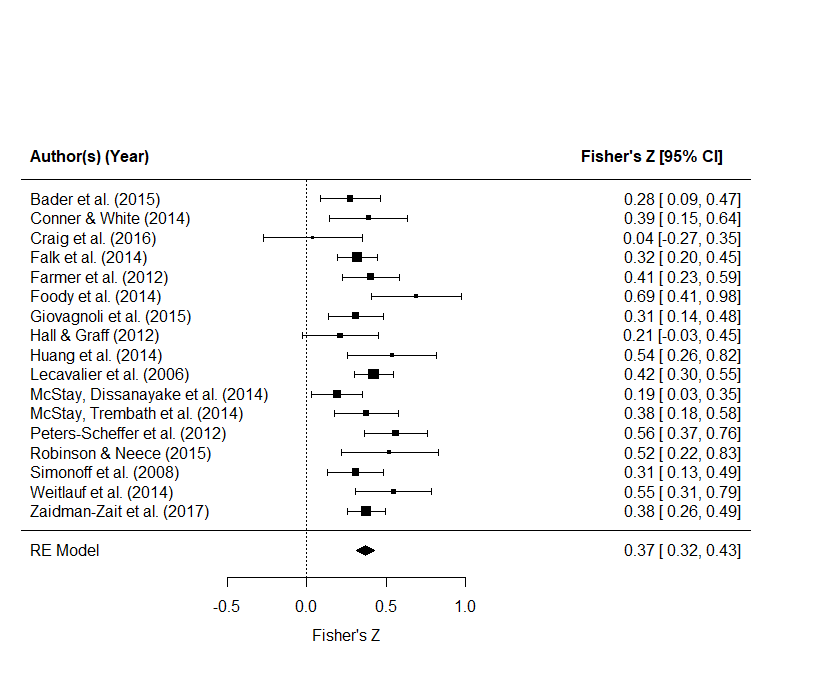


Supplementary Figure 2. Forest plot for Analysis 2 (associations between parenting stress and child externalizing)


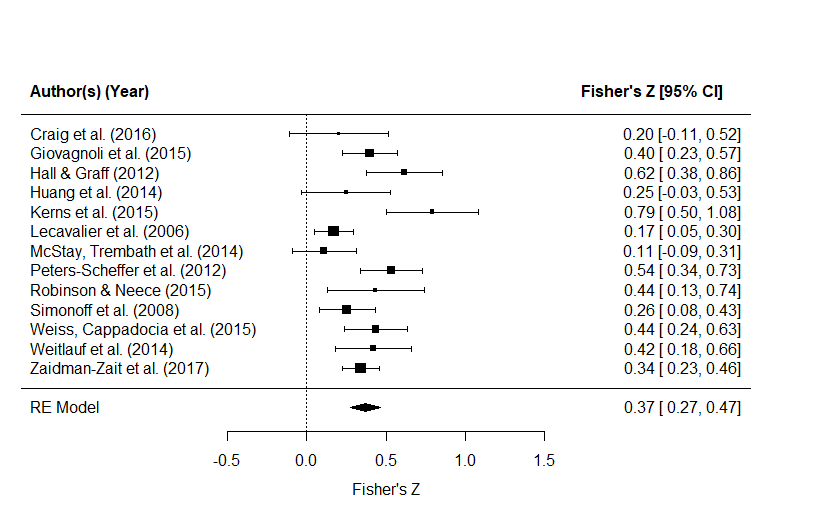


Supplementary Figure 3. Forest plot for Analysis 3 (associations between parenting stress and child internalizing)


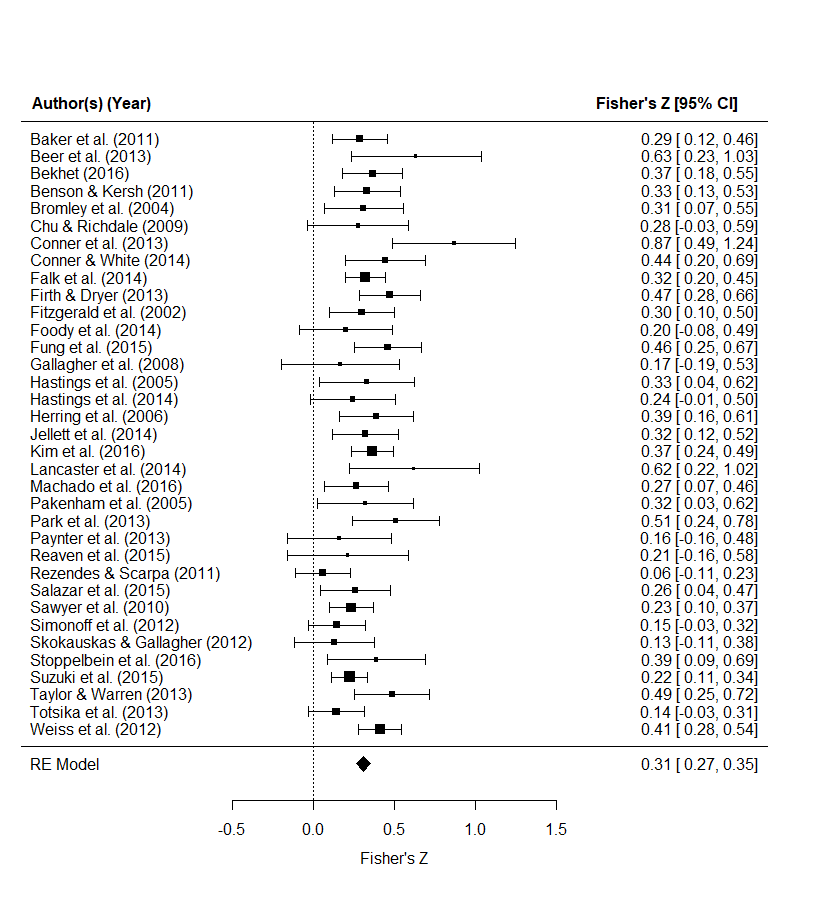


Supplementary Figure 4. Forest plot for Analysis 4 (associations between parent mental health problems and child total emotional and behavioral problems)


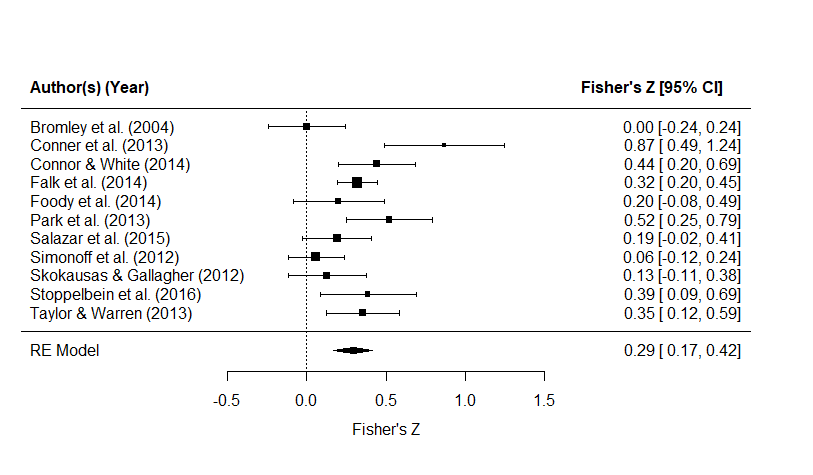


Supplementary Figure 5. Forest plot for Analysis 5 (associations between parent mental health problems and child externalizing)


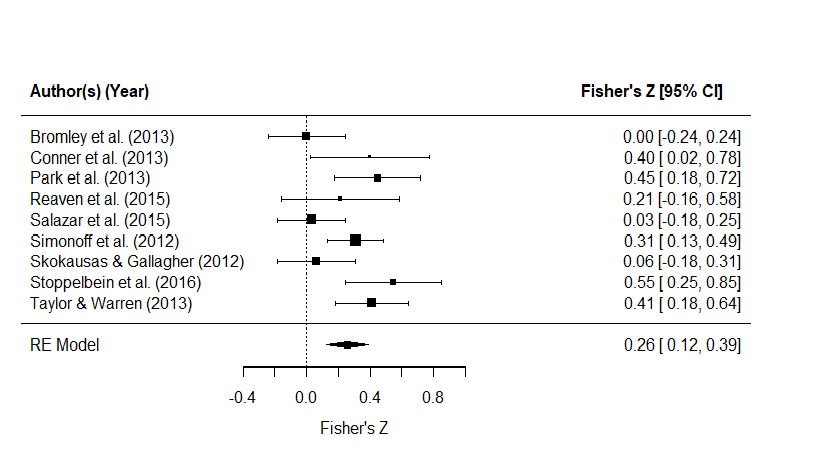


Supplementary Figure 6. Forest plot for Analysis 6 (associations between parent mental health problems and child internalizing)


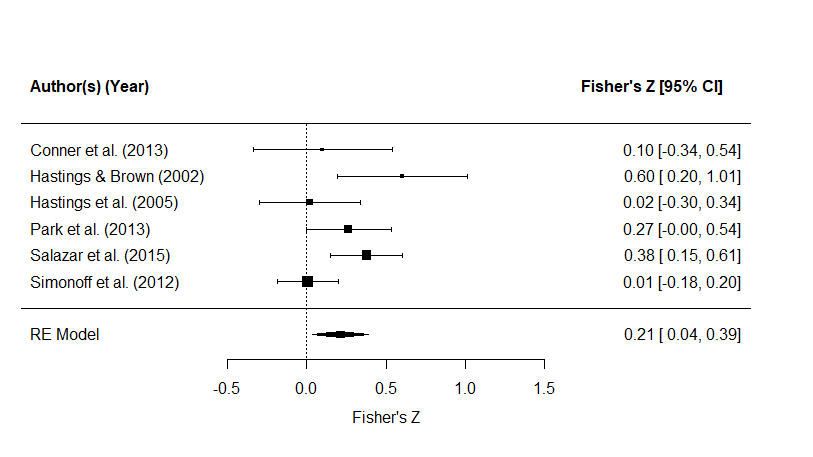


Supplementary Figure 7. Forest plot for Analysis 7 (associations between parent mental health problems and child total emotional and behavioral problems rated by alternative informant)


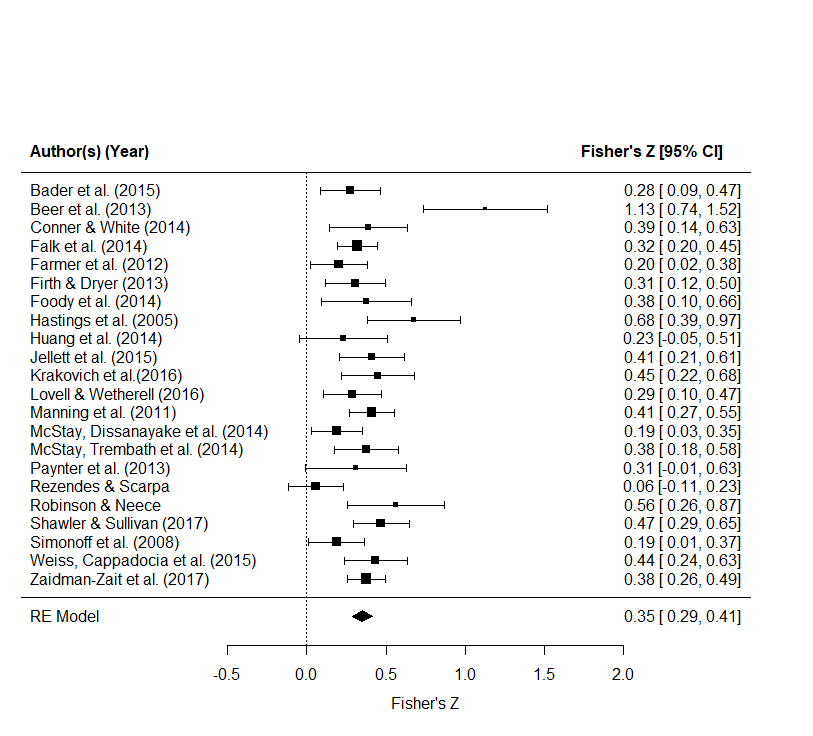


Supplementary Figure 8. Forest plot for Analysis 8 (associations between refined measures of parenting stress and child total emotional and behavioral problems).
